# Supplementary material for: vEMstitch: an algorithm for fully automatic image stitching of volume electron microscopy
Source: Gigascience. 2024 Oct 26;13:giae076. doi: 10.1093/gigascience/giae076 (PMC11512480; doi:10.1093/gigascience/giae076)

GigaScience

vEMstitch: an algorithm for fully automatic image stitching of volume electron microscopy

--Manuscript Draft--

Manuscript Number:	GIGA-D-24-00096
Full Title:	vEMstitch: an algorithm for fully automatic image stitching of volume electron microscopy
Article Type:	Research
Funding Information:	National Key Research and Development Program of China (2021YFF0704300, 2020YFA0712401) — Not applicable<br>National Natural Science Foundation of China projects Grant (62072280, 61932018, 62072441, 31730023, 32371248, 31521002) — Not applicable<br>Chinese Academy of Sciences (XDB37010100) — Not applicable<br>National Laboratory of Biomacromolecules of China (2019KF07) — Not applicable

Abstract:

Background

As software and hardware have developed, so has the scale of research into volume electron microscopy (vEM), leading to ever-increasing resolution. Usually, data collection is followed by image stitching: the same area is subjected to high-resolution imaging with a certain overlap, then the images are stitched together to achieve ultrastructure with large scale and high resolution simultaneously. However, there is currently no perfect method for image stitching, especially when the global feature distribution of the sample is uneven and the feature points of the overlap area cannot be matched accurately, which results in ghosting of the fusion area.

Results

We have developed a novel algorithm called vEMstitch to solve these problems, aiming for seamless and clear stitching of high-resolution images. In vEMstitch, the image transformation model is constructed as a combination of global rigid and local elastic transformation using weighted pixel displacement fields. Specific local geometric constraints and feature re-extraction strategies are incorporated to ensure that the transformation model accurately and completely reflects the characteristics of biological distortions. To demonstrate the applicability of vEMstitch, we conducted thorough testing on simulated datasets involving different transformation combinations, consistently showing promising performance. Furthermore, in real data sample experiments, vEMstitch successfully gives clear ultrastructure in the stitching region, reaffirming the effectiveness of the algorithm.

Conclusion

vEMstitch serves as a valuable tool for large-field and high-resolution image stitching. The clear stitched regions facilitate better visualization and identification in volume EM analysis. The source code is available at https://github.com/HeracleBT/vEMstitch.

Corresponding Author:	Bintao He<br>Shandong University<br>Qingdao, Shandong CHINA
Corresponding Author Secondary Information:	
Corresponding Author's Institution:	Shandong University
Corresponding Author's Secondary	

Powered by Editorial Manager® and ProduXion Manager® from Aries Systems Corporation

Institution:	
First Author:	Bintao He
First Author Secondary Information:	
Order of Authors:	Bintao He
	Yan Zhang
	Zhenbang Zhang
	Yiran Cheng
	Fa Zhang
	Fet Sun
	Renmin Han
Order of Authors Secondary Information:	
Additional Information:	
**Question**	**Response**
Are you submitting this manuscript to a special series or article collection?	No
**Experimental design and statistics**<br><br>Full details of the experimental design and statistical methods used should be given in the Methods section, as detailed in our Minimum Standards Reporting Checklist. Information essential to interpreting the data presented should be made available in the figure legends.<br><br>Have you included all the information requested in your manuscript?	Yes
**Resources**<br><br>A description of all resources used, including antibodies, cell lines, animals and software tools, with enough information to allow them to be uniquely identified, should be included in the Methods section. Authors are strongly encouraged to cite Research Resource Identifiers (RRIDs) for antibodies, model organisms and tools, where possible.<br><br>Have you included the information requested as detailed in our Minimum Standards Reporting Checklist?	Yes

Powered by Editorial Manager® and ProduXion Manager® from Aries Systems Corporation

**Availability of data and materials**<br><br>All datasets and code on which the conclusions of the paper rely must be either included in your submission or deposited in publicly available repositories (where available and ethically appropriate), referencing such data using a unique identifier in the references and in the "Availability of Data and Materials" section of your manuscript.<br><br>Have you have met the above requirement as detailed in our Minimum Standards Reporting Checklist?	Yes

Manuscript Click here to access/download;Manuscript;Manuscript.pdf

vEMstitch: an algorithm for fully automatic image stitching of volume electron microscopy

Bintao He[1], Yan Zhang[2], Zhenbang Zhang[3], Yiran Cheng[1], Fa Zhang[4], Fei Sun[2] and Renmin Han[1]

[1]*the Research Center for Mathematics and Interdisciplinary Sciences, Shandong University, Shandong 266000, China.*
[2]*the Center for Biological Imaging, Institute of Biophysics, Chinese Academy of Sciences, Beijing, 100190, China.*
[3]*School of Computer Science and Technology, Shandong University, Shandong 266000, China.*
[4]*School of Medical Technology, Beijing Institute of Technology, Beijing, 100190, China.*

Co-first-author: Bintao He, Yan Zhang, Zhenbang Zhang
Co-corresponding author: Renmin Han
the Research Center for Mathematics and Interdisciplinary Sciences, Shandong University,
Shandong 266000, China
hanrenmin@sdu.edu.cn;
Co-corresponding author: Fei Sun
Institute of Biophysics, Chinese Academy of Sciences,
Beingjing, China, 100101
feisun@ibp.ac.cn;

Abstract

Background:
As software and hardware have developed, so has the scale of research into volume electron microscopy (vEM), leading to ever-increasing resolution. Usually, data collection is followed by image stitching: the same area is subjected to high-resolution imaging with a certain overlap, then the images are stitched together to achieve ultrastructure with large scale and high resolution simultaneously. However, there is currently no perfect method for image stitching, especially when the global feature distribution of the sample is uneven and the feature points of the overlap area cannot be matched accurately, which results in ghosting of the fusion area.

Results:
We have developed a novel algorithm called vEMstitch to solve these problems, aiming for seamless and clear stitching of high-resolution images. In vEMstitch, the image transformation model is constructed as a combination of global rigid and local elastic transformation using weighted pixel displacement fields. Specific local geometric constraints and feature re-extraction strategies are incorporated to ensure that the transformation model accurately and completely reflects the characteristics of biological distortions. To demonstrate the applicability of vEMstitch, we conducted thorough testing on simulated datasets involving different transformation combinations, consistently showing promising performance. Furthermore, in real data sample experiments, vEMstitch successfully gives clear ultrastructure in the stitching region, reaffirming the effectiveness of the algorithm.

Conclusion:
vEMstitch serves as a valuable tool for large-field and high-resolution image stitching. The clear stitched regions facilitate better visualization and identification in volume EM analysis. The source code is available at `https://github.com/HeracleBT/vEMstitch`.

Keywords: Volume EM, serial section EM, image stitching, local distortion correction

1. Introduction

The continuous development of volume electron microscopy (vEM) in recent decades has led to considerable developments in life science, enabling rich structural information to be observed and captured at nanometer, micrometer, or even millimeter scale, such as in cells and tissues. However, although the ultimate goal in vEM is to achieve both large scale and high resolution, these are irreconcilable contradictions in the process of technological development. Usually when the magnification is relatively high, multiple images are taken in the target area; by moving the electron beam, each image is acquired with a certain overlap area (usually 10%), then the complete high-resolution image of the target region is obtained via image stitching Szeliski et al. (2007); Saalfeld (2019); Peddie and Collinson (2014); Peddie et al. (2022). Unfortunately, cut marks, folds, deformations, and breakage during sample preparation pose challenges and difficulties in imaging data processing Jin and Li (2015); Kaynig et al. (2010); Saalfeld (2019), and if the overlap areas happen to contain such artifacts, then accurate image stitching is almost impossible.

Currently, there are two types of image stitching in common use: (i) calculating the translation relationships among overlapping regions by using phase correlations in Fourier space Argyriou and Vlachos (2006); Tasdizen et al. (2010); Preibisch et al. (2009); Chalfoun et al. (2017); Wetzel et al. (2016) or (ii) calculating the geometric relationships among overlapping regions based on feature-based matching of the overlapping images Brown and Lowe (2007); Chow et al. (2006); Saalfeld et al. (2010); Khairy et al. (2018). The advantage of using phase correlations in Fourier space is rapid calculations, but if the images have obvious rotation or non-rigid deformation, then ideal seamless stitching is not possible with this method. Therefore, using phase correlations alone cannot provide large scale and high resolution in vEM-based microscopy, such as serial section scanning or transmission electron microscopy and serial block-face scanning electron microscopy Horstmann et al. (2012); Titze and Genoud (2016); Peddie and Collinson (2014); Peddie et al. (2022).

Feature-based approaches use distinctive local features Lowe (2004); Bay et al. (2008); Rublee et al. (2011); Ke and Sukthankar (2004) within images to identify matching patterns in adjacent tiles and then establish the corresponding relationships among features to estimate the transformation parameters. Unlike Fourier-based methods, which rely on relationships among global frequency components, feature points (FPs) generally reflect more-local properties of images, and this makes such methods more suitable and flexible for complex deformations. In the feature-based architecture, the predetermined transformation model and corresponding methods for extracting FPs are constantly being optimized Gao et al. (2011); Zaragoza et al. (2013); Lin et al. (2015); Li et al. (2018, 2019); Lin et al. (2011). An accurate transformation model captures composite transformation or deformations efficiently and reduces the parameter search space. Also, accurately matched FPs enhance the parameter estimation process, so a well-extracted set of FPs is key for accurate stitching by feature-based methods. However, biological samples have complex and changeable ultrastructure, and when its global feature distribution is inhomogeneous or the local information is overconcentrated and the structure information in other regions is sparse, then feature matching is ineffective, resulting in incorrect matching or even being unable to find the FPs (as shown in Figure 1). In this case, simple feature matching cannot achieve an accurate seamless mosaic, thereby affecting the registration among the stitched serial images in the next step of vEM image processing.

Herein, we present a feature-based stitching pipeline named vEMstitch, which is designed specifically for vEM images with uneven feature distribution and local deformation. vEMstitch aims to stitch input images seamlessly while correcting local distortion. Our approach uses a combined global-rigid–local-elastic model to depict complex transformations in vEM images. To estimate global transformation, we use local geometrical properties and random sample consensus (RANSAC) Fischler and Bolles (1981) to establish accurate corresponding FPs and calculate rigid parameters. In addressing local elastic transformations, we implement feature re-extraction to achieve a balanced distribution of FPs and thin-plate spline (TPS) functions to fit local distortion. Finally, the composite models are built as smoothed pixel displacement fields to generate seamless mosaics (Fig. 2). To validate the efficiency and robustness of vEMstitch, we generate three simulated datasets of different transformation combinations to quantify the accuracy. Furthermore, our experiments on real-world data also yield promising results. In summary, vEMstitch is superior to existing open-source stitching tools in generating seamless mosaics.

2. Methods

We propose a pipeline for accurate stitching of vEM images that combines global rigid transformation and local elastic correction. Our method is efficient at solving the misalignment and ghosting caused by local distortion of biological samples, specifically with large-field imaging inputs. As shown in Fig. 2, the pipeline comprises the following three main components: (i) geometry consistency constraints and RANSAC-based global-rigid estimation; (ii) FP distribution balance and local elastic correction based on TPS interpolation Bookstein (1989); (iii) combined global and local transformation and final mosaic generation.

2.1. Global-Rigid Estimation

Abundant FPs are effective for capturing the main characteristics of images, and accurate point-set matching helps with reliable identification of similar regions in input images. For two input images, we extract scale-invariant feature transform (SIFT) FPs and then match them coarsely by means of the closest two points ratio. Large-field images of biological specimens usually have massive and locally intensive small structures, resulting in many false matches. To acquire accurate corresponding points, we remove local

outliers to reduce the number of candidate points, then we use RANSAC based on geometry consistency constraints to determine the correct matching. Finally, the closed-form rigid parameters are calculated from the above filtered FP set.

2.1.1. Filtering of Local Outliers

Effective deformation estimation is based on accurate corresponding FP pairs. Compared to global transformation, local elastic deformation considering all matching points is more sensitive to outliers. The local outlier filtering involves two main stages: (i) filter the outliers coarsely on a per-image basis to reduce the number of candidate points; (ii) filter finely by the distance between matching points.

In the first stage, we consider the local distribution of FPs. A reliable point should not be isolated locally, and multiple adjacent points are better for depicting changes in a small region. In elastic transformation in particular, a locally isolated point of false matching easily results in discontinuous estimated pixel displacement. We define the average axial distance between source point i and each adjacent point in a window as w_i, and the filtering threshold $r^i_{adjacent}$ is

$$r^i_{adjacent} = \lambda_{adjacent} \frac{1}{N_i} \sum_j w_j \tag{1}$$

where point j is in the window of point i and N_i is the number of adjacent points.

In the second stage, we study the relative distances between matching points. The displacement or deformation of an organism is locally smooth, indicating that the corresponding points of adjacent FPs are usually located closely. Similar to the first stage, we define the Euclidean distance between source point i and the corresponding one as m_i, and the filtering threshold $r^i_{matching}$ is

$$r^i_{matching} = \lambda_{matching} \frac{1}{N_i} \sum_j m_j \tag{2}$$

where point j is in the window of point i and N_i is the number of adjacent points.

2.1.2. RANSAC Based on Local Geometric Constraints

Biological deformations are usually small and smooth, making it almost impossible to produce folding in small areas. Therefore, regarding FPs, the lines between matching points cannot cross in a local region. Furthermore, from a geometrical perspective, a pair of matching points should have the same geometric location in the neighborhood. To use the above geometric property simply and effectively, we propose a strategy combined with RANSAC for maintaining the relative positions of points. As shown in Fig. 3, given any three matched pairs, we determine the position of one point relative to the others by simple vector products.

The position judgment is then embedded into RANSAC to obtain a well-matched point set by the following procedure.

1. Input the matching FP sets X, Y of the two images and normalize them.

2. Randomly select four point pairs and calculate their respective vector products V_n, V_{n+1}, the signs of which are used to determine whether the position relationships of the raw and corresponding points are consistent. If any point does not satisfy the condition, skip this iteration.

3. Compute the closed-form homography parameters using good points, and calculate the projection error for each point. The inner points are defined as those for which the corresponding projection error is smaller than a given threshold ϵ.

4. Calculate steps 2 and 3 circularly until the given number of random sampling (in practice, $\sim 10^3$ is enough). Finally, return the well-matched sets with the most inner points.

2.1.3. Estimation of Closed-form Rigid Parameters

Having acquired well-matched points X, Y, we calculate closed-form rigid parameters using singular value decomposition (SVD) as follows:

$$\begin{cases} S = XY^T, \\ U\Sigma V = S, \\ R = VU^T, \\ t = u - Rv, \end{cases} \tag{3}$$

where $U\Sigma V$ is the SVD matrix, R is the rotation matrix, and t is the translation vector.

2.2. Local Deformation Correction

Local deformation estimation relies heavily on the extracted FPs and cannot work on regions without control points. Meanwhile, more-abundant feature information leads to more-accurate local distortion correction. However, in larger biological specimens of tissues or organs, the FPs are usually relatively sparse or even missing, and to tackle this problem, we propose feature re-extraction to enrich the extracted points and ensure that they are distributed well on all the overlapping regions. TPS functions based on the above points are then estimated to generate local pixel displacement fields, and the complete pipeline is shown in Fig. 4.

2.2.1. Image Enhancement and Feature Re-extraction

It may be difficult to extract features from raw images for various reasons, so an effective image enhancement method is necessary. First, we split an overlapping region into some small areas and check the number of FPs to determine where to re-extract features. We then use the Laplacian of Gaussian (LoG) to extract edge information, but the LoG image may be discontinuous, which is a considerable obstacle for later feature extraction. Therefore, we smooth the results using a simple weighted fusion strategy to obtain enhanced images:

$$fusion_i = Gau_i(I) * \alpha + loG(I) * (1 - \alpha), \tag{4}$$

where $Gau(\cdot)$ denotes the Gaussian blurring operation and $loG(\cdot)$ denotes the LoG operator.

The enhanced images are dominated by edge information, so we apply the corner-detection algorithm FAST Rosten and Drummond (2006) to the enhanced region to obtain the key points, and we use the ORB algorithm to assign orientations for each key point. After that, the fine point-set matching is the same as in Section 2.1.2.

2.2.2. Computation of Displacement Fields

TPS interpolation is a two-dimensional (2D) non-rigid transformation method that is commonly used in biological deformation estimation. Here, we use matching features as the control points in TPS interpolation to compute local pixel displacement fields, as shown in Fig. 5. Although local outliers are specifically processed in Section 2.1.1, the other matching points may contain incorrect relationships, and the TPS algorithm takes all input points as effective control ones. For improved robustness and stability, we use multiple iterations to further eliminate the outliers Li et al. (2018) as follows.

1. Solve the TPS linear equation to obtain the weights of each function component.
2. Collect the number of outliers as defined by the three-sigma criterion, and reject them if the number is larger than the given threshold.
3. Recalculate using the updated parameters to obtain the outliers, and repeat the above process.
4. Obtain a good set of control points to solve the TPS linear equation.

2.3. Image Blending

To extend local deformation smoothly from overlapping to non-overlapping regions, we propose a fusion strategy that effectively combines the global-rigid and local elastic transformations. A seamless mosaic is then generated by linearly weighting the overlapping regions.

2.3.1. Generation of Smooth Pixel Displacement Field

To transition the local deformation field smoothly to the global-rigid transformation, we design a smooth pixel displacement field. The weight η of the pixel displacement field is used to adjust the smoothness of the deformation; η ranges between 0 and 1 so that the deformation function of the overlapping region decreases gradually to zero. As given by (5), if the distance between the current position and the boundary of the overlapping region is greater than ε_1, then η is 1, and if it is less than ε_0, then η is 0:

$$\eta = \begin{cases} \frac{\varepsilon_1 - dis_i}{\varepsilon_1 - \varepsilon_0} & (\varepsilon_0 < dis_i < \varepsilon_1), \\ 1 & (\varepsilon_0 > dis_i), \\ 0 & (\varepsilon_1 < dis_i), \end{cases} \tag{5}$$

where dis_i is the maximum distance of the pixel point (u, v) from the boundary of the local deformation field.

This keeps the values of the deformation field inside the local deformation field constant and eliminates those outside the local deformation field, while the values in the transition region are obtained by weighted averaging. This achieves a smooth combination of global-rigid transformation and local elastic correction, i.e.,

$$f_s(x, y) = \eta f(x, y). \tag{6}$$

2.3.2. Generation of Blending Masks

To obtain a linear weighting mask, we begin by smoothing the deformed image masks $mask_1$ and $mask_2$ linearly so that the mask value of the overlap region $mask_{overlap}$ decreases gradually from 1 to 0. The overlap of the final blending masks $mask'_1$ and $mask'_2$ is set to $mask_{overlap}$. Finally, we use the smoothed mask to weight blend the images I'_1 and I'_2 processed by the pixel shift field to obtain the final stitched image I_{res}:

$$mask_{overlap} = \begin{cases} 0 & (m + r < x < d), \\ \frac{x-m+r}{2r} & (m - r < x < m + r), \\ 1 & (u < x < m - r), \end{cases} \tag{7}$$

where m represents the middle position of the overlapping region, u and d are the lower and upper ends of the overlapping region, respectively, and the radius $r = (d - m) * \alpha$, with the parameter α set to 0.15, and

$$I_{res} = I'_1 * mask'_1 + I'_2 * mask'_2. \tag{8}$$

3. Results

To evaluate the proposed method quantitatively and visually, we applied it to three simulated datasets constructed with different transformation combinations, and also some real-world datasets. The evaluations included comparing the proposed method with three open-source stitching tools, i.e., the Fourier-based stitching tools Fiji Schindelin et al. (2012); Abràmoff et al. (2004) and MIST Chalfoun et al. (2017) and the feature-based stitching method Trakem2 Saalfeld et al. (2010). These open-source tools are available as ImageJ/Fiji plugins.

3.1. Simulation Experiments

To quantify the similarity among overlapping regions and the discrepancy among rigid parameters, we developed a simulation flow to construct datasets of different transformations. We selected 100 raw images of size 3072 × 3072 from the Circuit Reconstruction from Electron Microscopy Images challenge (https://cremi.org/), then to simulate mechanical displacement, we applied random rigid transformations $\mathcal{R}_i$ to the raw image I_0 with parameters (t_i, r_i) (a rotation angle r_i of $\pm[0.5°, 1°]$, and a translation t_i from [0.5%, 1%] times the height or width of the raw image, respectively) and obtained I_i $(i = 2, 3, 4)$. We then generated pixel displacement matrices to mimic biological deformation by iteratively adding Gaussian smoothed random displacement, and the sampling grids were applied on I_i $(i = 2, 3, 4)$ to generate deformed images $\overline{I}_i$ $(i = 2, 3, 4)$. Finally, four tiles were cut with an overlap rate of 10% to generate the final image grid. The total process of data simulation can be summarized as follows:

$$I_i = \mathcal{R}_i(I_0), i = 2, 3, 4, \tag{9}$$

$$\begin{aligned}\overline{I}_i &= E_i(D_{final}) \\ &= E_i(\sum_{i=1}^{N}[\alpha \cdot Gauss(D^i_{rand}, \sigma)], I_i), i = 2, 3, 4,\end{aligned} \tag{10}$$

where D^i_{rand} is the ith randomly generated displacement field with elements in the range of $[-1, 1]$, Gauss$(\cdot)$ is a 2D Gaussian filter operator that is applied separately on the two channels of D^i_{rand}, α determines the magnitude of the displacement, and σ controls the smoothness of the deformation. In our experiments, we used $\alpha = 0.08 \cdot \text{size}(I_0)$, $\sigma = 2 \cdot \text{size}(I_0)$, and $N = 4$.

We conducted three simulation experiments, i.e., experiments I, II, and III, each of which had its own transformation combination. In experiment I, we applied only random rigid transformation (only translation, without rotation) to the raw images to simulate the translation of samples. In experiment II, we applied random rigid transformation (translation and rotation) to the raw images to simulate the rigid movement of samples. In experiment III, we applied both random rigid transformation (translation and rotation) and random elastic transformation to the raw images to simulate composite image deformation.

3.2. Results for Simulated Data

We quantify the stitching accuracy of the final mosaic using the following metrics. To evaluate the similarity between two overlapping regions, we use NCC and SSIM (structural similarity) Wang et al. (2004) defined as follows:

$$NCC(x, y) = \frac{\sigma_{xy}}{\sigma_x \sigma_y},$$

$$SSIM(x, y) = \frac{(2\mu_x\mu_x + C_1)(2\sigma_{xy} + C_2)}{(\mu_x^2 + \mu_y^2 + C_1)(\sigma_x^2 + \sigma_y^2 + C_2)},$$

where μ_x and μ_y are the average values of images x and y, σ_x and σ_y are the standard deviations, σ_{xy} is the covariance, and C_1 and C_2 are constants. To measure the differences between the ground-truth rigid parameters and the estimated ones, we use the translation error and rotation error of each tile.

We summarize the average metrics of the simulated datasets in Table 1 and choose three representative examples as shown in Fig. 7. To the right of the stitching results are some partial enlargements of the overlapping regions with the corresponding NCC and SSIM values.

In experiment I, which involved only translation deviation, all the stitching tools including both the Fourier-based and feature-based methods achieved nearly perfect metrics. This is supported by the visual representations in Fig. 7 (I), where the overlapping regions in all the stitching mosaics are the same as the ground truth. These results show that both Fourier-based and feature-based methods can perform well on simple translation estimation. In experiment II with rigid transformation, Table 1 shows clearly that feature-based methods (ours and TrakEM2) outperform Fourier-based ones (Fiji and MIST) across all quantitative metrics. Fourier-based methods struggle with rotation estimation and exhibit unacceptable translation errors in cases with complex transformation. By contrast, feature-based TrakEM2 presents better metrics and the rotation estimation error is acceptable for stitching, but the translation error is not sufficiently stable. As shown in Fig. 7 (II), TrakEM2's stitching results produce obvious folding when subjected to larger translation errors. By contrast, our method consistently performs the best and shows robustness.

Table 1: Average Stitching Metrics on Simulation Datasets

Method		Metric			
		Overlapping region		Transformation parameters	
		NCC	SSIM	Translation error	Rotation error
I	Fiji	0.989 ± 0.007	0.938 ± 0.039	0.202 ± 0.046	-
	Mist	0.994 ± 0.002	0.959 ± 0.014	0.305 ± 0.088	-
	TrakEM2	0.969 ± 0.0951	0.921 ± 0.220	1.115 ± 4.590	0.022 ± 0.211
	Ours	**0.999** ± **0.001**	**0.989** ± **0.003**	**0.010** ± **0.003**	**0**
II	Fiji	0.529 ± 0.136	0.228 ± 0.113	7.118 ± 1.411	-
	Mist	0.267 ± 0.199	0.180 ± 0.148	113.989 ± 105.244	-
	TrakEM2	0.983 ± 0.078	0.951 ± 0.171	1.636 ± 4.223	0.012 ± 0.013
	Ours	**0.990** ± **0.003**	**0.999** ± **9e-05**	**0.875** ± **0.196**	**0**
III	Fiji	0.456 ± 0.125	0.189 ± 0.088	8.699 ± 2.016	-
	Mist	0.211 ± 0.172	0.134 ± 0.121	108.984 ± 101.742	-
	TrakEM2	0.865 ± 0.076	0.444 ± 0.139	5.467 ± 4.053	0.012 ± 0.011
	Ours	**0.998** ± **0.001**	**0.957** ± **0.018**	**5.135** ± **1.758**	**0.006** ± **0.003**

A key contribution to this is that we use local geometry information to establish more-accurate feature matching. In experiment III involving both rigid displacement and elastic distortion, Table 1 shows the superior performance of feature-based methods (ours and TrakEM2) over Fourier-based ones (Fiji and MIST) in all metrics. Fourier-based methods cannot handle complex composite transformations, and their estimated translation errors are unacceptable in such cases. Feature-based TrakEM2 and our method both have larger parameter errors than those in experiment II. This is attributed to the challenge of local distortion, which can cause correct FP pairs to be falsely rejected in the RANSAC process. However, our approach uses all filtered FP pairs to model deformation effectively by using a combination of global-rigid and local elastic techniques. The highest NCC and SSIM values of our method in Table 1 further indicate its effectiveness in local distortion correction. Moreover, Fig. 7 (III) shows the representative stitching results from the comparison methods, which exhibit obvious ghosting, whereas our method produces a clear and seamless mosaic.

3.3. Results for Real-world Data

To illustrate further the feasibility and effectiveness of our method, we show three examples of 3×3 micrograph stitching (a common application of vEM imaging) provided by the Center for Biological Imaging, Institute of Biophysics, Chinese Academy of Sciences. The three examples of mussels settling in the deep sea were imaged by scanning electron microscopy after resin embedding at normal temperature and frozen ultrathin slicing. The accelerating voltage of the microscope was set to 2 kV, and the detector was one involving concentric backscattered electrons. Fig. 8 shows the different mussel sections and the corresponding stitched results of Fiji, MIST, TrakEM2, and our method. In real-world sections, the overlapping rate is ca. 10% and translation transformation plays the main role, but some rotation and local distortion are inevitable. Therefore, the Fourier-based Fiji and MIST and the feature-based TrakEM2 all exhibit obvious ghosting or folding, and more severely in some areas with less valid information, such as the cell membrane. Because limited pixel information prevents accurate parameter estimation, vEMstitch enriches the image by feature re-extraction and achieves the best stitching results, as the enlargements indicate. Meanwhile, in some areas with rich information, the Fourier-based Fiji and MIST exhibit blurred overlapping regions, and the feature-based TrakEM2 exhibits insufficient displacement estimation and obvious visible boundaries. Excessive FPs make mismatches more difficult to filter, so vEMstitch uses local geometry properties to reduce the number of candidate points and establish more-accurate point matching. Generally, the NCC values of selected enlargements of Fiji, MIST, and TrakEM2 are mostly between 0.6 and 0.8 or even lower than 0.1. Neglecting the effects of local deformation, the existing stitching tools are inadequate for clear and seamless mosaic generation of large-field serial section electron microscopy images.

Also, in Table 2 we assess the stitching quality using the three aforementioned real datasets, each of which comprised 200 sets of 3×3 tiles. Because of the absence of ground-truth transformation, we mainly evaluate the overlapping regions of adjacent tiles

Table 2: Average Stitching Metrics on Real Datasets

		NCC	SSIM
Sample I	Fiji	0.52±0.16	0.02±0.02
	Mist	0.47±0.22	0.02±0.02
	TrakEM2	0.70±0.19	0.08±0.12
	Ours	**0.94±0.04**	**0.25±0.11**
Sample II	Fiji	0.79±0.08	0.13±0.05
	Mist	0.79±0.13	0.16±0.08
	TrakEM2	0.89±0.05	0.26±0.10
	Ours	**0.97±0.02**	**0.50±0.10**
Sample III	Fiji	0.69±0.07	0.11±0.06
	Mist	0.71±0.08	0.15±0.07
	TrakEM2	0.89±0.05	0.24±0.09
	Ours	**0.97±0.01**	**0.52±0.10**

using the NCC and SSIM metrics. In the experiments, it is evident that feature-based methods consistently outperform Fourier-based approaches such as Fiji and MIST. Actually, regions with NCC values below 0.7 often exhibit obvious ghosting, and the lower NCC values produced by Fourier-based methods indirectly indicate the presence of nonlinear distortion in the tiles. By contrast, our pipeline consistently gives the highest NCC values across all datasets, thereby emphasizing its robust distortion-correcting capabilities that ensure superior performance in seamless stitching.

3.4. Analysis of Modules

Proposed herein is a feature-based stitching architecture for large-field vEM images, and we focus mainly on two characteristics of FPs: matching accuracy and spatial distribution.

3.4.1. Module for Feature Matching

Accurate parameter estimation is dominated by the precision of feature matching. A commonly used and straightforward criterion in natural image processing involves comparing the nearest-neighbor and second-nearest-neighbor distances and filtering when this ratio is lower than a given threshold Lowe (2004). In fact, the ratio is high for features that are not distinctive, and lowering the threshold ratio enhances the accuracy of feature matching but often results in fewer retained feature pairs. In Fig. 9(a), we show how the precision and the number of acquired feature pairs change as the threshold ratio is increased, using the simulated datasets I, II, and III. Experiments I and II involve only rigid transformations (translation and rotation), and straightforward feature matching is effective for handling these cases while retaining hundreds of FP pairs. It performs well with a threshold ratio of 0.6, and the local outlier filtering and local geometry constraints proposed herein merely elevate the acceptable threshold ratio to ca. 0.8. Nevertheless, in experiment III with interference from local distortions, the description of features is less accurate than in experiments I and II, and thus the matching accuracy fails to improve as the threshold ratio is decreased. This reveals the limitations of straightforward feature matching, which reaches a bottleneck. The local outlier filtering strategy enhances the matching accuracy by ca. 10%, and local geometry constraints achieve an accuracy exceeding 80%, deemed acceptable for subsequent processes. Note that these two proposed strategies are based on initial matching results with an accuracy higher than 60%, and they cannot be applied to outcomes with lower accuracy or unmatched points. For better illustration, we also show a visual example in Fig. 9(b). The local outlier filtering strategy is effective at removing most points not located in the overlapping regions. Furthermore, local geometry constraints help to identify false matches by checking whether lines drawn between corresponding points intersect. As indicated by the red arrows in the third image, false FP matching occurs when both points lie on the edges of a spot. This situation leads easily to misjudgment and interferes with the subsequent local distortion correction.

3.4.2. Module for Feature Re-extraction

Feature-based algorithms rely heavily on the extracted FPs, particularly when addressing local distortion correction. In the context of vEM images, the inherent characteristics of specimens can lead to certain regions having few or even no FPs after the

matching process. Fig. 10 shows this phenomenon clearly, typically in the edge area of the organism. Within the complex and diverse internal structures of the organism, an abundance of points can be found, providing adequate information for distinct FP extraction. However, near the organism's edges, the structures are often simple yet occasionally blurred, leading to insufficient FPs. These FPs are always lacking in distinction and may subsequently be filtered during the matching process. To illustrate this further, we show a distribution of points using a 50-pixel-wide counting box below the image. The feature re-extraction strategy is designed to extract more key points on the edge region or linear structure, by edge enhancement and image fusion. Meanwhile, the re-extraction strategy acts on only the selected regions not the whole overlapping area. Consequently, compared to feature matching at the global stage, the matching process in the re-extraction stage is considerably simpler and results in a larger number of accurate FP pairs, as shown in Fig. 10(b). To provide quantitative insights, Table 3 gives the average number of FPs from the real datasets, with the data again organized using a 50-pixel-wide counting box. Note that in Table 3, "ratio" is the threshold ratio in the matching process, "avg_fea_num" is the average number of raw FP pairs, "avg_fea_area" is the average number in regions with few FPs, and "avg_enhanced_area" is the average number in the area after applying the re-extraction strategy. As can be seen, the re-extraction strategy increases the number of FP pairs substantially.

Table 3: Average Number of FPs in Counting Boxes

ratio	avg_fea_num	avg_fea_area	avg_enhanced_area
0.2	3.41	1.33	2.26
0.3	15.11	3.27	8.71
0.4	41.64	3.75	14.33
0.5	87.06	3.83	19.44
0.6	147.96	3.88	26.82

Note that the proposed strategies were designed specifically for biological vEM data. The sections have a relatively fixed overlap rate, making local outlier filtering particularly effective because most matched points are located within belt-shaped regions. Meanwhile, because we are dealing with sections imaged by the same device and parameters, as opposed to natural images, there is no need to consider parallax or view-related issues. This simplifies the feature-matching process significantly, allowing us to confidently adopt rigid transformations as the best and only choice for global transformation. Furthermore, the distortions present in these sections are typically small, localized, unrelated to the structure, and sometimes even difficult to notice visually. Our proposed geometry constraints are aimed at preventing folding and severe local changes, meeting the inherent requirements. Besides, the unbalanced FP distribution is a direct consequence of the high-resolution biological structures of the specimens. These areas are concentrated primarily along the edges of the organism, thereby driving the development of our image edge enhancement strategy.

3.5. Robustness Analysis

3.5.1. Robustness to Noisy Inputs

In real-world scenarios, it is usual to encounter electron microscopy images with varying degrees of background noise, which can interfere with the alignment of overlapping regions. Table 4 summarizes the average stitching metrics on three noise-affected simulation datasets, corresponding to the datasets in Section 3.1. During this simulation, we kept images I_1 and I_4 clean and added different levels of additional noise (Gaussian noise) to images I_2 and I_3. Interestingly, with increasing noise level, the translation and rotation errors do not change significantly, which indicates the robustness of SIFT in noisy images and highlights the broad adaptability in rigid transformation estimation. Moreover, the consistently high values of NCC (all exceeding 0.9) show that our method remains effective even when working with noisy inputs.

3.5.2. Robustness to Deformation

Excessive local distortion definitely poses challenges for feature-based architecture. To assess the robustness of our method under different levels of simulated distortion, we conducted tests with varying iteration number N for displacement accumulation, as discussed in Section 3.1. To ensure that the deformation increased with increasing N, we fixed the random-number seeds for the displacement-field generation and then added them repeatedly. Fig. 11(a) presents the NCC and SSIM values for our method both with and without local distortion correction as the iteration number N increases. The yellow curves represent the cases where only rigid transformations are considered, resulting in low NCC values of less than 0.7 and extremely low SSIM values. This observation shows that the obvious local distortion exists in the overlapping regions. By contrast, the blue curves represent our method, which consistently yields high NCC values exceeding 0.9, indicating the success of our local distortion correction. Fig. 11(b) provides illustrative examples for $N = 0, 2, 4, 6,$ and 8, and fortunately micrographs exhibiting local deformations similar to those for $N = 2$ and 4 are more common in practical applications.

Table 4: Average Stitching Metrics on Noise-affected Simulation Datasets

	Noise (σ)	0.0	5.0	10.0	15.0	20.0
I	NCC	0.999 ± 0.001	0.988 ± 0.003	0.958 ± 0.010	0.915 ± 0.017	0.865 ± 0.022
	SSIM	0.989 ± 0.003	0.841 ± 0.035	0.662 ± 0.053	0.525 ± 0.056	0.425 ± 0.053
	Translation error	0.010 ± 0.003	0.015 ± 0.006	0.026 ± 0.010	0.037 ± 0.016	0.054 ± 0.028
	Rotation error	0	0	0	0	0
II	NCC	0.990 ± 0.003	0.990 ± 0.002	0.978 ± 0.006	0.954 ± 0.013	0.923 ± 0.021
	SSIM	0.999 ± 0.000	0.863 ± 0.033	0.703 ± 0.052	0.578 ± 0.058	0.482 ± 0.058
	Translation error	0.875 ± 0.196	0.876 ± 0.199	0.877 ± 0.195	0.878 ± 0.197	0.884 ± 0.198
	Rotation error	0	0	0	0	0
III	NCC	0.998 ± 0.001	0.993 ± 0.002	0.977 ± 0.006	0.953 ± 0.013	0.923 ± 0.021
	SSIM	0.957 ± 0.018	0.836 ± 0.041	0.682 ± 0.054	0.561 ± 0.059	0.467 ± 0.058
	Translation error	5.135 ± 1.758	5.123 ± 1.767	5.111 ± 1.749	5.159 ± 1.787	5.265 ± 1.889
	Rotation error	0.006 ± 0.003	0.006± 0.003	0.006± 0.004	0.006± 0.004	0.006± 0.004

4. Conclusion

vEMstitch is an accurate and robust 2D grid-based image stitching tool that is effective at handling both simple rigid displacement and complex local distortion. Its demonstrated accuracy and visual performance make vEMstitch more applicable for large-field and high-resolution images with possible composite deformations, as in vEM applications. Although vEMstitch emphasizes local distortion correction, the use of local geometric information also makes global-rigid estimation more accurate and robust. In terms of stitching accuracy and seamlessness, vEMstitch has broader applicability and stronger deformation modeling ability compared to existing open-source tools.

Despite the effectiveness of the proposed pipeline, vEMstitch has several limitations that should be noted. It was designed for grid images with a given scanning path, and the stitching order is determined in advance, so currently vEMstitch cannot process unordered data. Also, its feature-based stitching architecture means that vEMstitch cannot be used on large datasets, ones whose mosaics comprise tens or hundreds of tiles, this being because the total run time would be unacceptable and much longer than with Fourier-based methods. However, the global-rigid estimation module of vEMstitch is independent of local distortion correction and could be used on a standalone basis; the simulation experiments reported in Section 3.1 also indicated the superiority of vEMstitch in rigid parameter estimation, and users could obtain less-accurate mosaics rapidly and robustly by bypassing the subsequent modules.

5. Availability of source code and requirements

Project name: vEMstitch
Project home page: https://github.com/HeracleBT/vEMstitch
Operating system(s): Platform independent
Programming language: Python
Other requirements: https://github.com/HeracleBT/vEMstitch/blob/main/environment.yaml
License: GNU GPL.

6. Data Availability

The source code, test examples and part of simulation results are available at https://github.com/HeracleBT/vEMstitch.

7. List of abbreviations

FP: feature points; RANSAC: random sample consensus; TPS: thin-plate spline; SIFT: scale-invariant feature transform; SVD: singular value decomposition; LoG: Laplacian of Gaussian; NCC: normalized cross-correlation; SSIM: structural similarity.

8. Competing interests

The authors declare they have no competing interests.

9. Funding

This work was supported by the National Key Research and Development Program of China [2021YFF0704300, 2020YFA0712401], National Natural Science Foundation of China projects Grant [62072280, 61932018, 62072441, 31730023, 32371248 and 31521002], the Chinese Academy of Sciences (CAS) [XDB37010100] and the National Laboratory of Biomacromolecules of China [2019KF07].

10. Authors' contributions

B.H.: Develop the method, construct the experiments, write and review the manuscript. R.H., F.Z.: Identify research goals and aims, provide research direction. F.S.: Explain the characteristics of the problem and provide technical support. Y.Z.: Write the Abstract and Introduction. Z.Z.: Write the draft of the section Methods. Y.C.: Implement and write the simulation experiments.

References

Abràmoff, M.D., Magalhães, P.J., Ram, S.J., 2004. Image processing with imagej. Biophotonics international 11, 36–42.

Argyriou, V., Vlachos, T., 2006. A study of sub-pixel motion estimation using phase correlation., in: BMVC, Citeseer. pp. 387–396.

Bay, H., Ess, A., Tuytelaars, T., Van Gool, L., 2008. Speeded-up robust features (surf). Computer vision and image understanding 110, 346–359.

Bookstein, F.L., 1989. Principal warps: Thin-plate splines and the decomposition of deformations. IEEE Transactions on pattern analysis and machine intelligence 11, 567–585.

Brown, M., Lowe, D.G., 2007. Automatic panoramic image stitching using invariant features. International Journal of Computer Vision 74, 59–73.

Chalfoun, J., Majurski, M., Blattner, T., Bhadriraju, K., Keyrouz, W., Bajcsy, P., Brady, M., 2017. Mist: Accurate and scalable microscopy image stitching tool with stage modeling and error minimization. Scientific Reports 7. doi:https://doi.org/10.1038/s41598-017-04567-y.

Chow, S.K., Hakozaki, H., Price, D.L., MacLean, N.A., Deerinck, T.J., Bouwer, J.C., Martone, M.E., Peltier, S.T., Ellisman, M.H., 2006. Automated microscopy system for mosaic acquisition and processing. Journal of microscopy 222, 76–84.

Fischler, M.A., Bolles, R.C., 1981. Random sample consensus: a paradigm for model fitting with applications to image analysis and automated cartography. Communications of the ACM 24, 381–395.

Gao, J., Kim, S.J., Brown, M.S., 2011. Constructing image panoramas using dual-homography warping, in: CVPR 2011, pp. 49–56. doi:10.1109/CVPR.2011.5995433.

Horstmann, H., Körber, C., Sätzler, K., Aydin, D., Kuner, T., 2012. Serial section scanning electron microscopy (s3em) on silicon wafers for ultra-structural volume imaging of cells and tissues. PloS one 7, e35172.

Jin, P., Li, X., 2015. Correction of image drift and distortion in a scanning electron microscopy. Journal of microscopy 260. doi:10.1111/jmi.12293.

Kaynig, V., Fischer, B., Müller, E., Buhmann, J.M., 2010. Fully automatic stitching and distortion correction of transmission electron microscope images. Journal of Structural Biology 171, 163–173. URL: https://www.sciencedirect.com/science/article/pii/S1047847710001401, doi:https://doi.org/10.1016/j.jsb.2010.04.012.

Ke, Y., Sukthankar, R., 2004. Pca-sift: A more distinctive representation for local image descriptors, in: Proceedings of the 2004 IEEE Computer Society Conference on Computer Vision and Pattern Recognition, 2004. CVPR 2004., IEEE. pp. II–II.

Khairy, K., Denisov, G., Saalfeld, S., 2018. Joint deformable registration of large em image volumes: A matrix solver approach. arXiv preprint arXiv:1804.10019 .

Li, J., Jiang, P., Song, S., Xia, H., Jiang, M., 2019. As-aligned-as-possible image stitching based on deviation-corrected warping with global similarity constraints. IEEE Access 7, 156603–156611. doi:10.1109/ACCESS.2019.2944852.

Li, J., Wang, Z., Lai, S., Zhai, Y., Zhang, M., 2018. Parallax-tolerant image stitching based on robust elastic warping. IEEE Transactions on Multimedia 20, 1672–1687. doi:10.1109/TMM.2017.2777461.

Lin, C.C., Pankanti, S.U., Ramamurthy, K.N., Aravkin, A.Y., 2015. Adaptive as-natural-as-possible image stitching, in: 2015 IEEE Conference on Computer Vision and Pattern Recognition (CVPR), pp. 1155–1163. doi:10.1109/CVPR.2015.7298719.

Lin, W.Y., Liu, S., Matsushita, Y., Ng, T.T., Cheong, L.F., 2011. Smoothly varying affine stitching, in: CVPR 2011, IEEE. pp. 345–352.

Lowe, D.G., 2004. Distinctive image features from scale-invariant keypoints. International Journal of Computer Vision 60, 91–110.

Peddie, C.J., Collinson, L.M., 2014. Exploring the third dimension: volume electron microscopy comes of age. Micron 61, 9–19.

Peddie, C.J., Genoud, C., Kreshuk, A., Meechan, K., Micheva, K.D., Narayan, K., Pape, C., Parton, R.G., Schieber, N.L., Schwab, Y., et al., 2022. Volume electron microscopy. Nature Reviews Methods Primers 2, 51.

Preibisch, S., Saalfeld, S., Tomancak, P., 2009. Globally optimal stitching of tiled 3d microscopic image acquisitions. Bioinformatics 25, 1463–1465.

Rosten, E., Drummond, T., 2006. Machine learning for high-speed corner detection, in: Computer Vision–ECCV 2006: 9th European Conference on Computer Vision, Graz, Austria, May 7-13, 2006. Proceedings, Part I 9, Springer. pp. 430–443.

Rublee, E., Rabaud, V., Konolige, K., Bradski, G., 2011. Orb: An efficient alternative to sift or surf, in: 2011 International conference on computer vision, Ieee. pp. 2564–2571.

Saalfeld, S., 2019. Chapter 12 - computational methods for stitching, alignment, and artifact correction of serial section data, in: Müller-Reichert, T., Pigino, G. (Eds.), Three-Dimensional Electron Microscopy. Academic Press. volume 152 of *Methods in Cell Biology*, pp. 261–276. URL: https://www.sciencedirect.com/science/article/pii/S0091679X19300585, doi:https://doi.org/10.1016/bs.mcb.2019.04.007.

Saalfeld, S., Cardona, A., Hartenstein, V., Tomančák, P., 2010. As-rigid-as-possible mosaicking and serial section registration of large sstem datasets. Bioinformatics 26, i57–i63.

Schindelin, J., Arganda-Carreras, I., Frise, E., Kaynig, V., Longair, M., Pietzsch, T., Preibisch, S., Rueden, C., Saalfeld, S., Schmid, B., et al., 2012. Fiji: an open-source platform for biological-image analysis. Nature methods 9, 676–682.

Szeliski, R., et al., 2007. Image alignment and stitching: A tutorial. Foundations and Trends® in Computer Graphics and Vision 2, 1–104.

Tasdizen, T., Koshevoy, P., Grimm, B.C., Anderson, J.R., Jones, B.W., Watt, C.B., Whitaker, R.T., Marc, R.E., 2010. Automatic mosaicking and volume assembly for high-throughput serial-section transmission electron microscopy. Journal of neuroscience methods 193, 132–144.

Titze, B., Genoud, C., 2016. Volume scanning electron microscopy for imaging biological ultrastructure. Biology of the Cell 108, 307–323.

Wang, Z., Bovik, A.C., Sheikh, H.R., Simoncelli, E.P., 2004. Image quality assessment: from error visibility to structural similarity. IEEE transactions on image processing 13, 600–612.

Wetzel, A.W., Bakal, J., Dittrich, M., Hildebrand, D.G., Morgan, J.L., Lichtman, J.W., 2016. Registering large volume serial-section electron microscopy image sets for neural circuit reconstruction using fft signal whitening, in: 2016 IEEE Applied Imagery Pattern Recognition Workshop (AIPR), pp. 1–10. doi:10.1109/AIPR.2016.8010595.

Zaragoza, J., Chin, T.J., Brown, M.S., Suter, D., 2013. As-projective-as-possible image stitching with moving dlt, in: 2013 IEEE Conference on Computer Vision and Pattern Recognition, pp. 2339–2346. doi:10.1109/CVPR.2013.303.

Fig. 1. Two common phenomena in feature-based stitching.

Fig. 2. Pipeline of proposed method. The input images are processed sequentially in three main modules: global-rigid estimation, local elastic correction, and image blending.

Fig. 3. Schematic of determining relative position. Local geometry constraints aim to preserve the relative spatial positions of matched points in RANSAC.

Fig. 4. Pipeline of local elastic correction. The core is the feature re-extraction strategy to balance the distribution of feature points (FPs).

Fig. 5. Sketch of thin-plate spline (TPS) interpolation. The red dots are the matching points of the blue ones.

Fig. 6. Workflow for generating simulated data. A random rigid transformation is applied to simulate the rigid movement of samples, and an optional random elastic transformation is applied on the overlapping regions to simulate local distortion.

Fig. 7. Comparison of stitched results for simulated data: (a) image sequence; (b) Fiji; (c) MIST; (d) ground truth; (e) TrakEM2; (f) our method.

Fig. 8. Real-world 3×3 mussel section data. The first column shows the input microscopy images.

Fig. 9. Feature-matching results: (a) curves of precision and feature number with increasing threshold ratio; (b) a typical visual example.

Fig. 10. Distribution of FPs: (a) point distribution without feature re-extraction; (b) point distribution with feature re-extraction.

Fig. 11. Overlapping regions evaluated for different deformation levels: (a) NCC and SSIM values for our method with or without local distortion correction; (b) examples for different deformation levels, showing local distortion becoming more obvious as N increases.

Figure1

Click here to access/download;Figure;Figure1.pdf

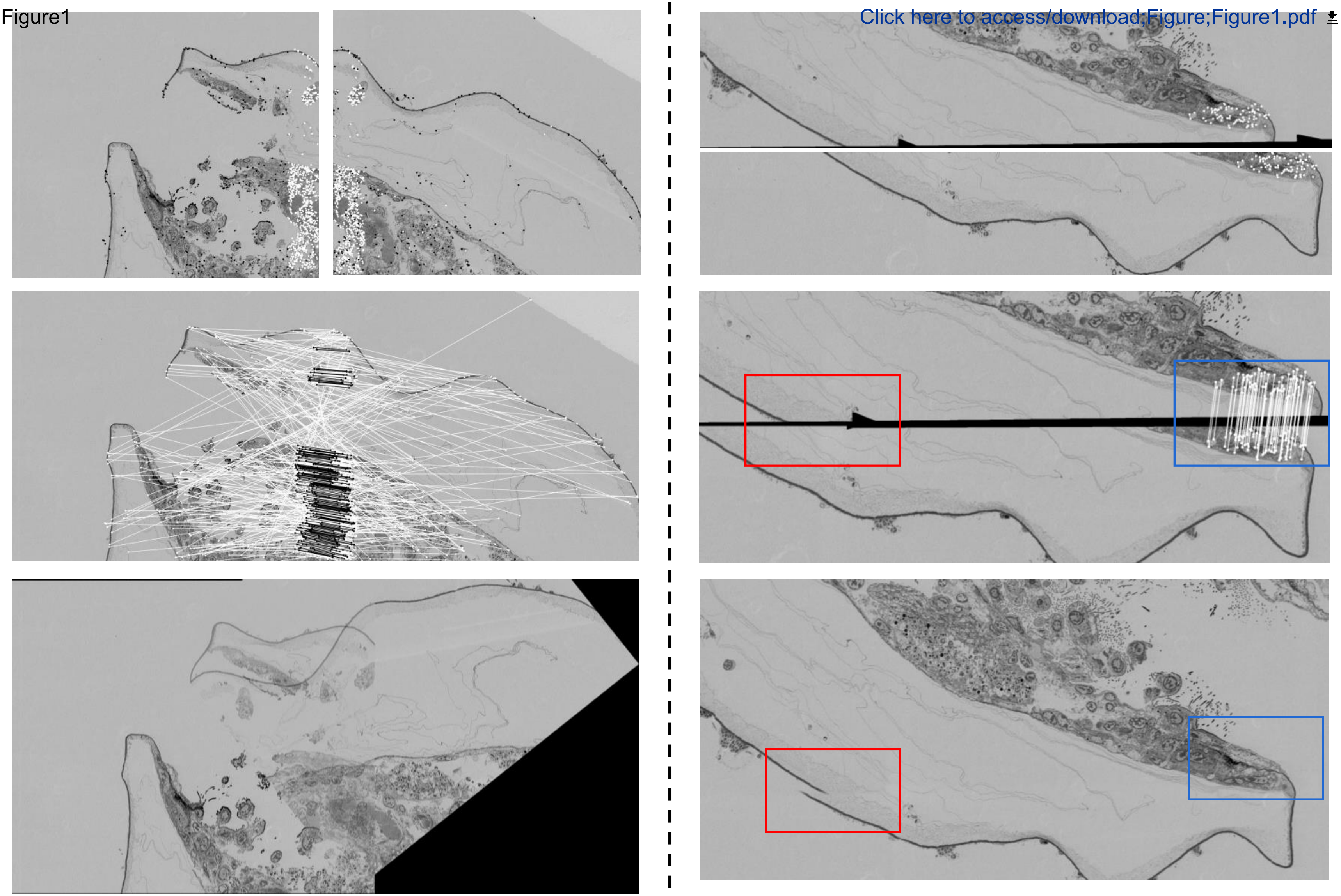

False feature matching(white line)

locally feature point missing(red)

Figure2 Click here to access/download;Figure;Figure2.pdf

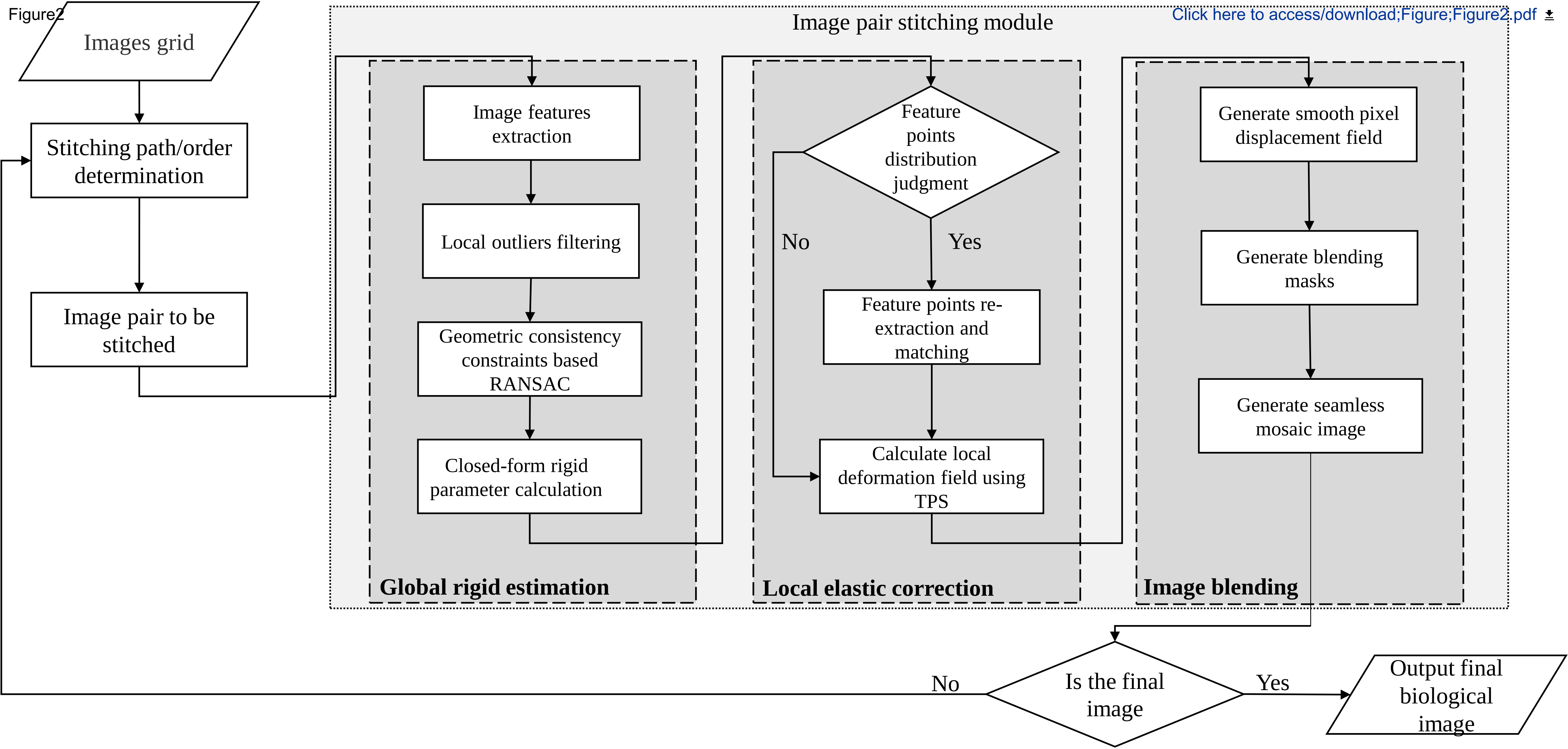

Figure3
Click here to access/download;Figure;Figure3.pdf

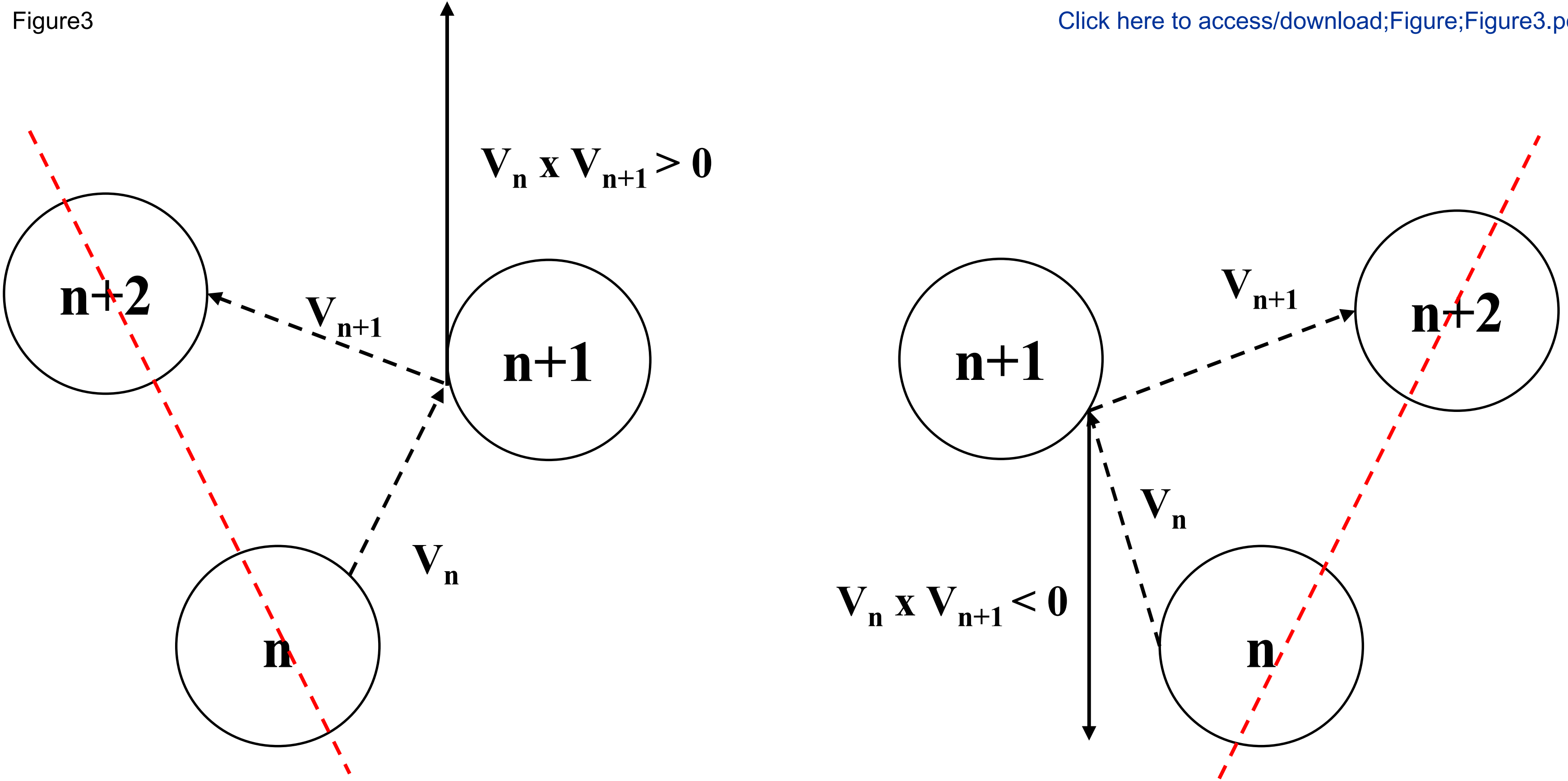

a) Situation when the cross product is positive **b) Situation when the cross product is negative**

Figure4

Click here to access/download;Figure;Figure4.pdf

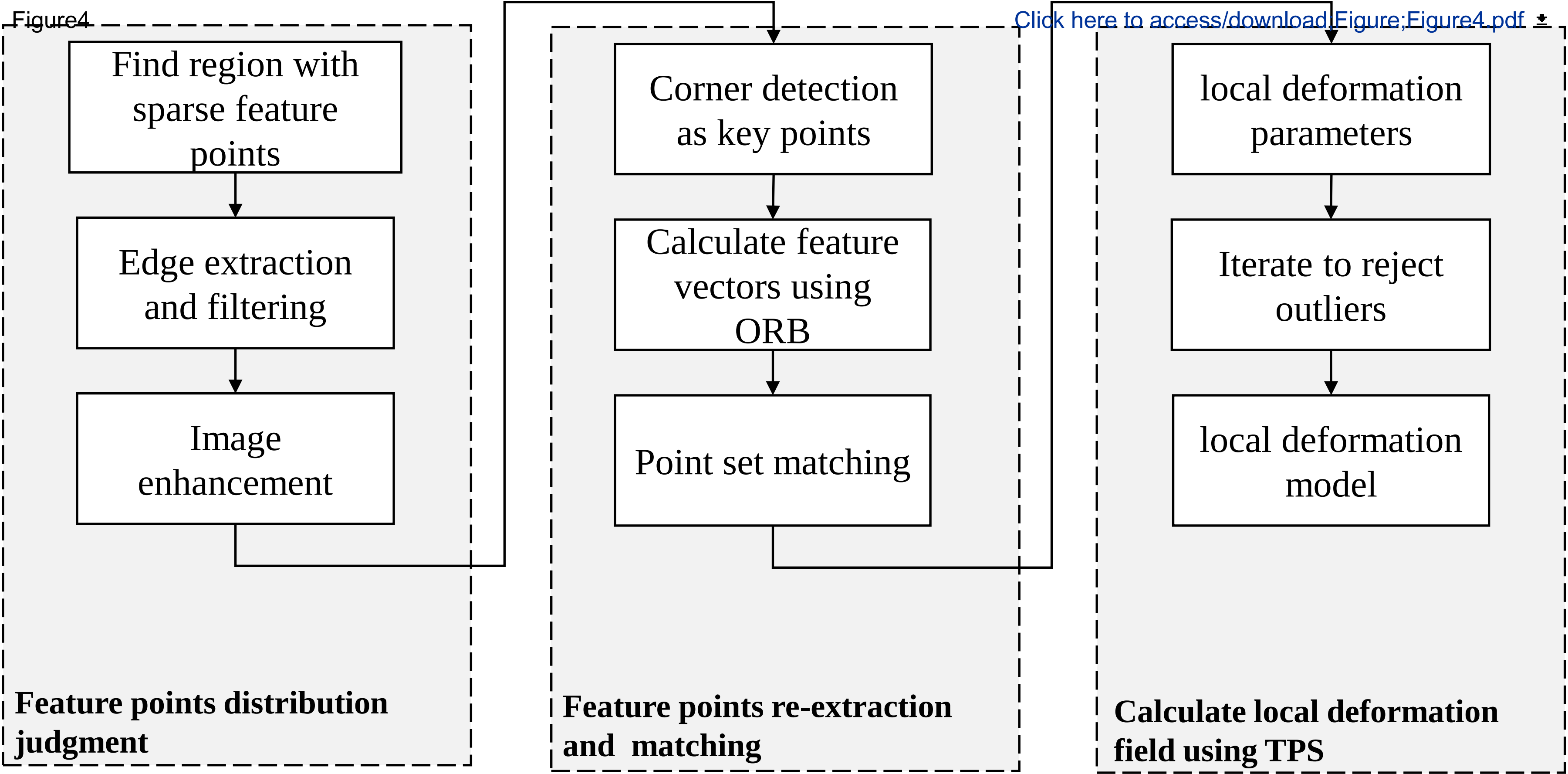

Figure5

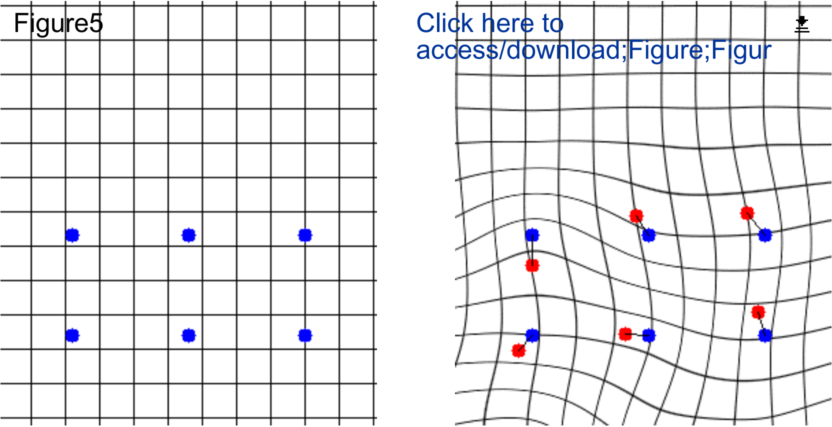

Click here to
access/download;Figure;Figur

Figure6

Click here to access/download;Figure;Figure6.pdf

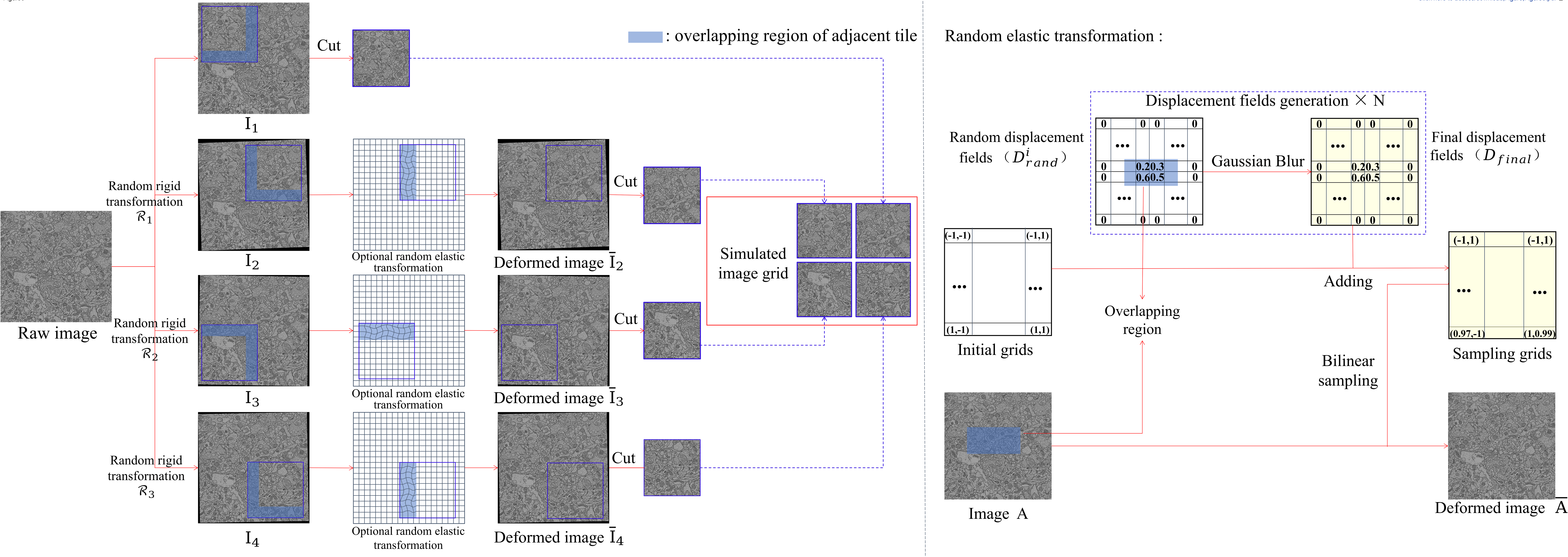

Figure7

Click here to access/download;Figure;Figure7.pdf

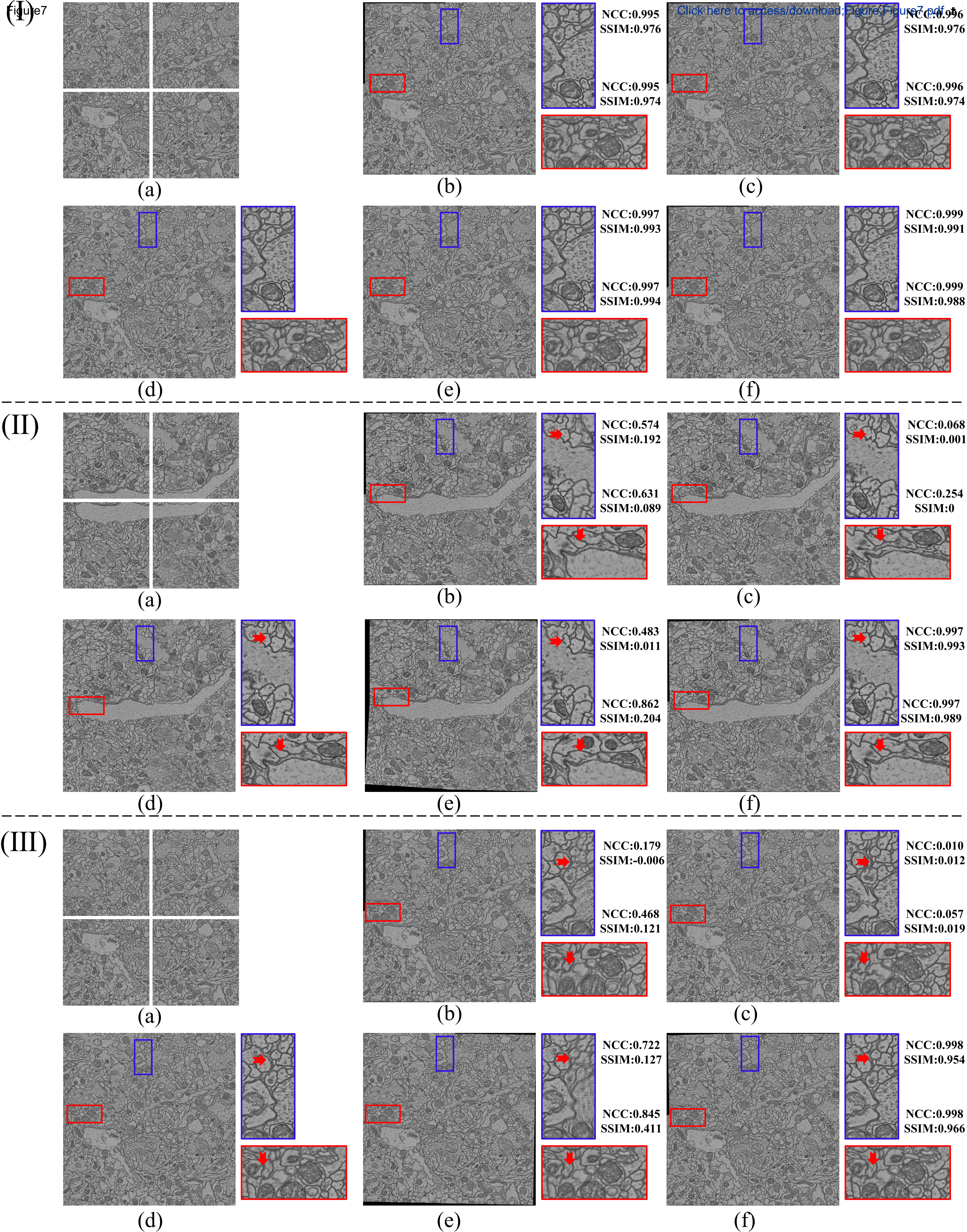

Figure8

Click here to access/download;Figure;Figure8.pdf

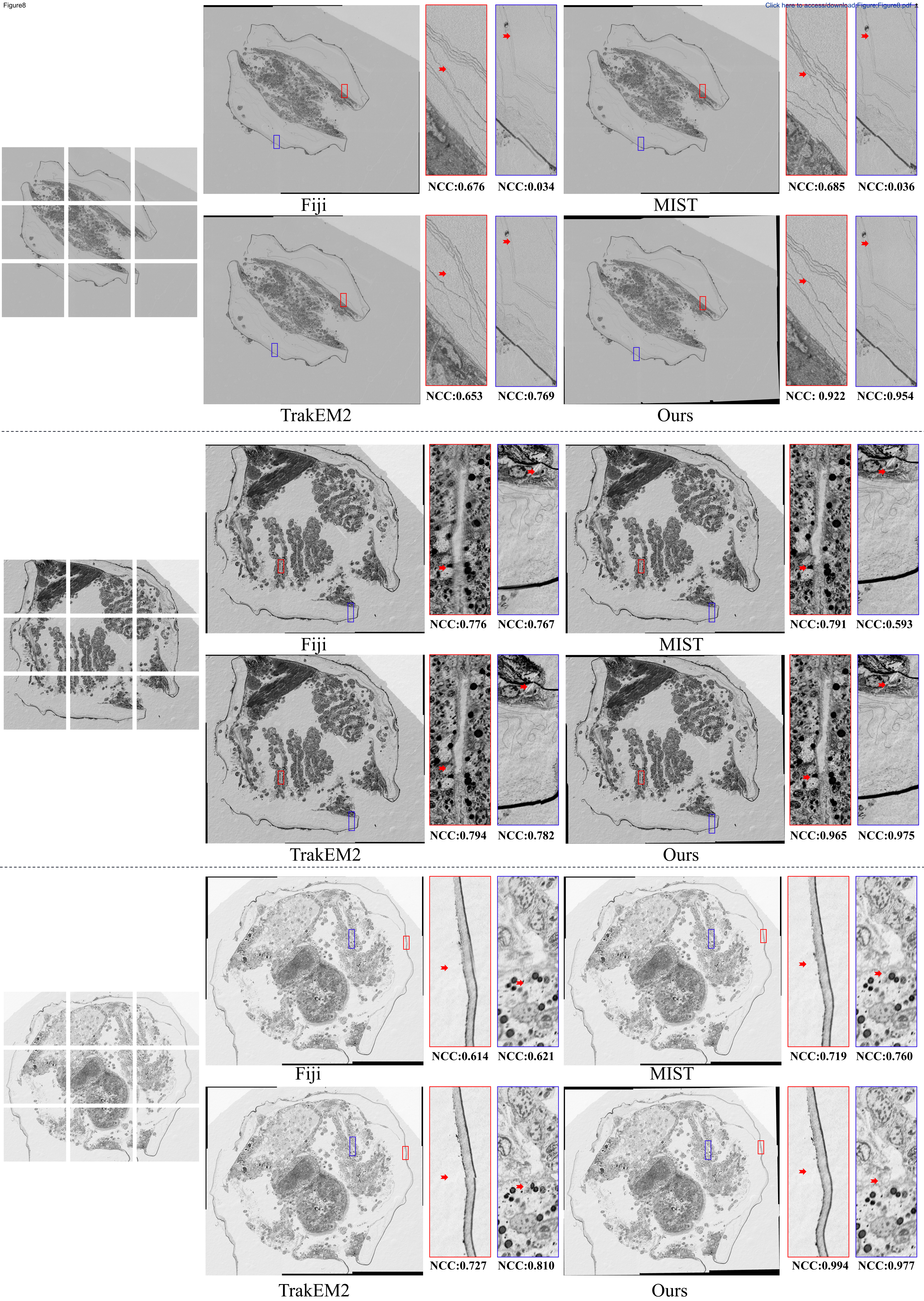

Figure9
Click here to access/download;Figure;Figure9.pdf

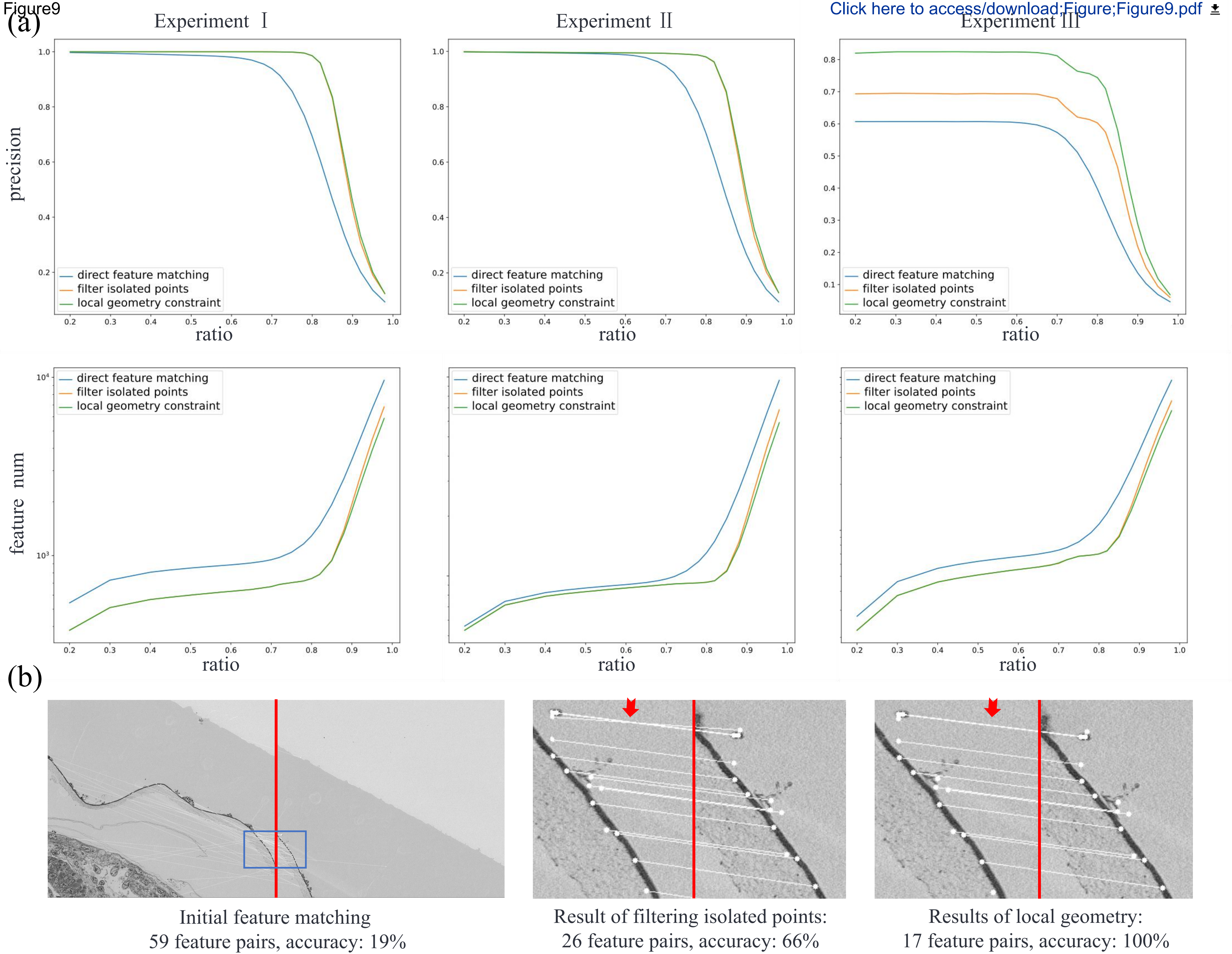

Figure10 Click here to access/download;Figure;Figure10.pdf

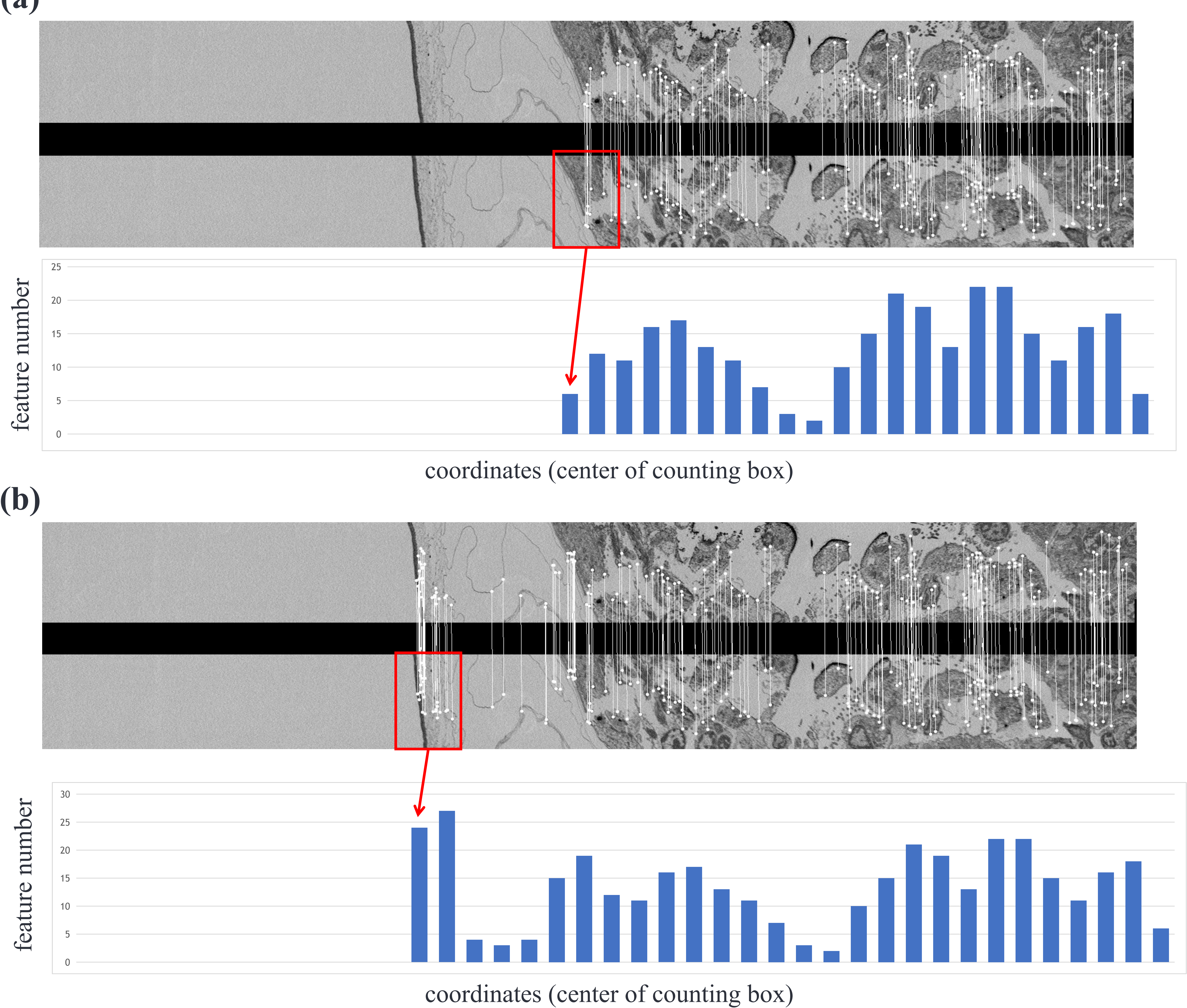

Figure11
Click here to access/download;Figure;Figure11.pdf

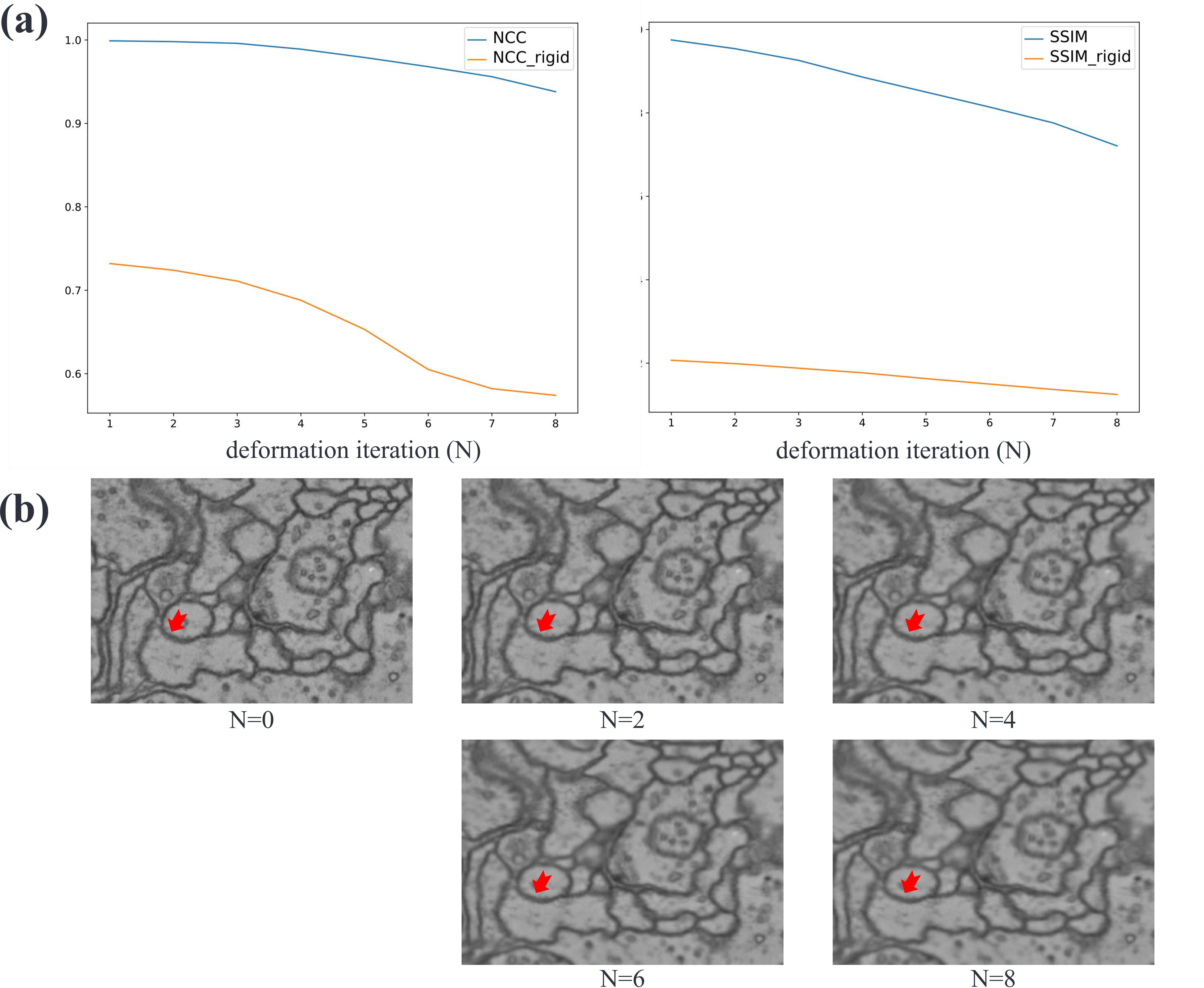

Supplement: giae076_GIGA-D-24-00096_Original_Submission [file giae076_giga-d-24-00096_original_submission.pdf]
