# Supplementary material for: vEMstitch: an algorithm for fully automatic image stitching of volume electron microscopy
Source: Gigascience. 2024 Oct 26;13:giae076. doi: 10.1093/gigascience/giae076 (PMC11512480; doi:10.1093/gigascience/giae076)
Supplement: giae076_Response_to_Reviewer_Comments_Original_Submission [file giae076_response_to_reviewer_comments_original_submission.pdf]

Author's Response To Reviewer Comments

The complete response letter is attached as a .pdf file (in supplement type).

To: Editors

Dear Sirs,
We are pleased to submit the revised manuscript GIGA-D-24-00096 titled “vEMstitch: an algorithm for fully automatic image stitching of volume electron microscopy”, by Bintao He, Yan Zhang, Zhenbang Zhang, Yiran Cheng, Fa Zhang, Fei Sun, and Renmin Han, to be considered as an article at GigaScience.

We really appreciate your and both reviewers' valuable feedback and suggestions, which have extremely helped us improve the quality and clarity of our work. Once again, we would like to express our sincere gratitude. We are delighted to have the opportunity to elevate the manuscript to the next level with the assistance and guidance of our esteemed reviewers. Therefore, we carefully considered all comments and made necessary changes to improve the quality and impact of our work. We believe that we have addressed the concerns raised by the reviewers to the best of our ability by either adding new results or clarification.

In response to the reviewers' feedback, we have made the following major changes in this revision:

1. We have updated a C++ implementation of vEMstitch in Github. Now both Python and C++ versions are open access, with Python version being more flexible and C++ version running faster. We temporarily met some issues in SIGH UP of bio.tools account, but we will provide the identifiers in the final draft.
2. For reviewer 1, we clearly clarify the application scope of vEMstitch. First, we pointed out that current volume EM techniques are not only used in neural circuit analysis, but also extended throughout the life science. vEMstitch aims to provide accurate and seamless 2D stitching for overall cell or organism imaging, where a section consists of tens of tiles, rather than hundreds or thousands of. Second, we emphasized that the development of vEMstitch was driven by real data stacks (mussels settling in the deep sea). These stacks contain 1543 sections and occupy 80GB storage. Fiji and TrakEM2, two widely used tools, fail to produce acceptable 2D mosaics. Finally, we compared the computational time, and demonstrated vEMstitch runs faster than TrakEM2 in the feature-based architecture.
3. We responded in detail to reviewer 1's question 2 and introduced how local distortions affect the final mosaic.
4. To answer reviewer 1's question 3, 6, 7, 11, we added the detailed description of some parameters and proved that default parameters were tested on different real data stacks.
5. We responded in detail to reviewer 1's question 4 and updated the previous Figure 9 (now Figure 12) in the main text by introducing pre-given overlapping rate.
6. We responded in detail to reviewer 1's question 5 and explained most of non-linear components are retained after relaxed RANSAC.
7. We responded in detail to reviewer 1's question 8 and provided visual examples of LoG edge extraction to demonstrate the necessary image fusion operation.
8. We added an experiment in the response letter to show the ORB description is sufficient for feature extraction in enhanced regions, considering balance between accuracy and speed, to answer reviewer 1’s question 9.
9. We responded in detail to reviewer 1's question 10 and added formula and description of TPS controlling in the main text section “Computation of Displacement Fields”. Large distortions are filtered in iterations to smooth pixel displacement fields.
10. We responded in detail to reviewer 1's question 12 and illustrated NCC and SSIM indirectly measure the alignment accuracy from global and local perspective, respectively.
11. We provided the parameter settings used in compared tools, and added some real examples in the response letter to justify the parameter combinations to answer reviewer 1’s question 13.
12. To answer reviewer 2’s suggestions 1 and 2, we added a Figure 1 to motivate the overall processing and some descriptions to expand the literature and infrastructure of vEM in the main text section “Introduction”.

13. We followed reviewer 2’s suggestion 3 and added a section “2D stitching vs. 3D alignment” in the main text to discuss the relationship between stitching and alignment in detail.
14. We followed reviewer 2’s suggestion 4 and provided corresponding p-values of paired t-tests on Table 1.
15. To answer reviewer 2’ s question 5, we added a section “Segmentation analysis” in the main text to prove that blurring and misalignment definitely affect the subsequent segmentation performance.
16. We followed reviewer 2’ s suggestion 6 and added a section “Visual Example for Ablation Study” in the main text to describe the target and result addressed by each step in the algorithm.
17. We followed reviewer 2’’s suggestion 7 and added a section “Runtime analysis” in the main text to show the faster feature-based stitching of vEMstitch than TrakEM2.

We believe that the revised manuscript represents a significant improvement over the initial submission and addresses all the issues raised by the reviewers. We are confident that it meets the high standards of quality and rigor expected by GigaScience.
