# Supplementary material for: vEMstitch: an algorithm for fully automatic image stitching of volume electron microscopy
Source: Gigascience. 2024 Oct 26;13:giae076. doi: 10.1093/gigascience/giae076 (PMC11512480; doi:10.1093/gigascience/giae076)
Supplement: giae076_Response_to_Reviewer_Comments_Revision_1 [file giae076_response_to_reviewer_comments_revision_1.pdf]

Author's Response To Reviewer Comments

To: Editors
Aug 7, 2024

Dear Sirs,
We are pleased to submit the revised manuscript GIGA-D-24-00096R1 titled “vEMstitch: an algorithm for fully automatic image stitching of volume electron microscopy”, by Bintao He, Zhenbang Zhang, Yan Zhang, Yiran Cheng, Fa Zhang, Fei Sun, and Renmin Han, to be considered as an article at GigaScience.

We really appreciate your and both reviewers' further valuable feedback and suggestions, which have extremely helped us improve the quality and clarity of our work again. Once again, we would like to express our sincere gratitude. We are delighted to have the opportunity to elevate the manuscript to the next level with the assistance and guidance of our esteemed reviewers. Therefore, we carefully considered all comments and made necessary changes to improve the quality and impact of our work. We believe that we have addressed the concerns raised by the reviewers to the best of our ability by either adding new results or clarification.

In response to the reviewers' feedback, we have made the following major changes in this revision:

1. We followed reviewer 1’s suggestion, and added the runtime of our Python implementation.
2. To answer reviewer 1’s question 2.1, we provided real-world examples to demonstrate that deformation is caused not only by microscope issues, but also by errors during sample preparation. We also clarified the meaning of technical term “radiation damage”.
3. We responded in detail to reviewer 1's question 2.2 and proved that the window size is suitable for most scenarios.
4. We followed reviewer 1’s suggestion 2.3 and distinguished the curves in Figure 10.
5. We responded in detail to reviewer 1's question 2.6 and explained that sufficiently accurate point correspondences can omit the effects of slight errors in pairwise stitching. We also provided a 5*5 stitching example to show our tool’s ability for larger sections.
6. We responded in detail to reviewer 1's question 2.7 and explained that LoG filtered images mainly pose challenges for later feature matching, not feature extraction.
7. To answer reviewer 1's question 2.8, we first explained that feature re-extraction is usually used in inter-row stitching, and unevenly distributed feature points mainly affect local regions, with a relatively minor impact on global rigid estimation. Second, we emphasized that local enhancement strategy is judged between global rigid estimation and local distortion correction. The Figures 2 and 4 were modified. Finally, we stated that very few points extracted by direct SIFT are hard to be correctly filtered and matched.
8. We provided some examples to show the different imaging properties between CREMI dataset and our real-world dataset. We further stated that the ablation study on simulated CREMI dataset cannot reflect the performance of our tool, and alternatively we enriched the step-wise real-world experiment in Figure 9.
9. We responded in detail to reviewer 1's final question and analyzed the similarities and differences between feature-based methods and phase-based approaches.
10. We followed reviewer 2’s suggestion 1 to add formulas for Dice coefficients and Hausdorff distances. We also added standard deviations and t-tests in Table 3.
11. Following reviewer 2’s suggestion 2, we combined previous Figures 7 and 8 (related to simulation experiments) and additionally added two small charts in Figure 6.
12. We followed reviewer 2’s suggestion 3 and carefully corrected some inappropriate words.

We believe that the revised manuscript represents a significant improvement over the initial submission and addresses all the issues raised by the reviewers. We are confident that it meets the high standards of quality and rigor expected by GigaScience.
Sincerely yours,
Bintao He

Research Center for Mathematics and Interdisciplinary Sciences, and Frontiers Science Center for Nonlinear

Expectations (Ministry of Education), Shandong University, Shandong 266000, China. E-Mail:hebintao@mail.sdu.edu.cn
