# Supplementary material for: vEMstitch: an algorithm for fully automatic image stitching of volume electron microscopy
Source: Gigascience. 2024 Oct 26;13:giae076. doi: 10.1093/gigascience/giae076 (PMC11512480; doi:10.1093/gigascience/giae076)
Supplement: giae076_Response_to_Reviewer_Comments_Revision_3 [file giae076_response_to_reviewer_comments_revision_3.pdf]

Author's Response To Reviewer Comments

To: Editors
Sep 18, 2024

Dear Sirs,
We are pleased to submit the revised manuscript GIGA-D-24-00096 titled “vEMstitch: an algorithm for fully automatic image stitching of volume electron microscopy”, by Bintao He, Zhenbang Zhang, Yan Zhang, Yiran Cheng, Fa Zhang, Fei Sun, and Renmin Han.

In response to your feedback, we have carefully corrected the section “References” and uploaded the Latex files to Editorial Manager.

We believe that it meets the high standards of quality and rigor expected by GigaScience.
Sincerely yours,
Bintao He

Research Center for Mathematics and Interdisciplinary Sciences, and Frontiers Science Center for Nonlinear Expectations (Ministry of Education), Shandong University, Shandong 266000, China E-Mail:hebintao@mail.sdu.edu.cn
