# Supplementary material for: vEMstitch: an algorithm for fully automatic image stitching of volume electron microscopy
Source: Gigascience. 2024 Oct 26;13:giae076. doi: 10.1093/gigascience/giae076 (PMC11512480; doi:10.1093/gigascience/giae076)
Supplement: giae076_Reviewer_1_Report_Original_Submission — Yuelong Wu -- 4/16/2024 Reviewed [file giae076_reviewer_1_report_original_submission.pdf]

Reviewer Report

Title: vEMstitch: an algorithm for fully automatic image stitching of volume electron microscopy

Version: Original Submission **Date:** 4/16/2024

Reviewer name: Yuelong Wu

Reviewer Comments to Author:

In this work, the authors described a new python package called vEMstitch for 2D montaging of volume electron microscopy data. The software relied on feature-based methods to establish corresponding landmarks within overlapping areas between neighboring image tiles, and then employed rigid models blended with local elastic refinements derived from thin-plate spline interpolation to obtain the transformations. The author compared the stitching qualities of their pipeline to those of Fiji, MIST and Trakem2 on a couple of simulated datasets as well as a small collection of real data and claimed better accuracy.The authors certainly proposed some interesting ideas, like combining a rigid transform with an elastic one by blending the transformation fields. However, I found the manuscript suffered a few major shortcomings.First, the author failed to demonstrate the applicability of their method to "large-scale" datasets, an important criterion for publication in Gigascience (according to the reviewer guidelines). There was no data provided regarding the speed and the scalability of their software. The datasets used in the manuscript were small in the context of recent development of volume EM. Indeed, in the conclusion, the author mentioned "vEMstitch cannot be used on large datasets, … because the total run time would be unacceptable".My second major critique concerns the writing style. While I could follow the manuscript's overall structure, I found that the detailed descriptions of the methodologies often lacked clarity and might benefit from some editing. The choice of words could lead to confusion in numerous places. Equations contained symbols without clear definitions.The following are specific questions/comments regarding certain sections of the text:- In the introduction, the authors stated that "if the overlap areas happen to contain such artifacts, then accurate image stitching is almost impossible". While I can understand that the artifacts may pose challenges to 3D alignment, how would that affect 2D stitching? Admittedly, there might be some secondary effects like beam charging accompanying the artifact, but for 2D stitching the microscope essentially scans the same image contents in the overlapping areas.- What are the Î» values in equation (1) and (2)? How were they determined? And how were the window sizes in those equations determined? Could the authors provide insights into the generalizability of these parameters across different datasets, especially if they required tuning?- About filtering of local outliers, from Figure 9 (b), it seems that the "filtering isolated points" step mostly filtered out the feature points outside the overlapping areas. Giving that the overlap ratio can typically be derived from metadata such as microscope stage coordinates, why was feature matching applied to the entire images rather than being restricted to the expected overlapping areas?- The matching points went through RANSAC, which assumed a linear model. That means the nonlinear component of the transformation might be potentially filtered out. Then how would these filtered points give rise to nonlinear deformation in later TPS interpolation step?- Was the term "inner points" intended to refer to "inliers"?- In equation (3), the definitions of u, v?- Could the author clarify the

meaning of "the LoG image may be discontinuous"?- The authors mentioned the usage of FAST as the detector and ORB (which also uses FAST as its detector) to assign orientations. Then what was used for the descriptors? Could the authors clarify the rationale behind using ORB features in this context, considering the previous use of SIFT features?- In section 2.2.2, how was the tuning parameter controlling the rigidity of the deformation determined in TPS interpolation? Also, could the author clarify the second step? What was measured as the basis to detect the outliers? The residue errors of the interpolation?- In equation (5), what is Îµ0, Îµ1. Also, could the author clarify what does equation (6) mean?- Could the authors provide the rationale of using SSIM in evaluating the stitching accuracy? The stitching is about the finding the accurate geometric transformations. Therefore, the luminance and contrast terms in SSIM are irrelevant here since they are all photometric measurements. That leaves us the structure term, which is essentially the NCC. So, in my opinion the two metrics are redundant.- Tools like FIJI or TrakEM2 have a lot of parameters that can be tuned to achieve good results. Could the authors provide detailed information how they conducted the experiments to compare the accuracies between different tools, and how they chose the parameters.

Level of Interest

Please indicate how interesting you found the manuscript: Choose an item.

Quality of Written English

Please indicate the quality of language in the manuscript: Choose an item.

Declaration of Competing Interests

Please complete a declaration of competing interests, considering the following questions:

- Have you in the past five years received reimbursements, fees, funding, or salary from an organisation that may in any way gain or lose financially from the publication of this manuscript, either now or in the future?
- Do you hold any stocks or shares in an organisation that may in any way gain or lose financially from the publication of this manuscript, either now or in the future?
- Do you hold or are you currently applying for any patents relating to the content of the manuscript?
- Have you received reimbursements, fees, funding, or salary from an organization that holds or has applied for patents relating to the content of the manuscript?
- Do you have any other financial competing interests?
- Do you have any non-financial competing interests in relation to this paper?

If you can answer no to all of the above, write 'I declare that I have no competing interests' below. If your reply is yes to any, please give details below.

I declare that I have no competing interests

I agree to the open peer review policy of the journal. I understand that my name will be included on my report to the authors and, if the manuscript is accepted for publication, my named report including any attachments I upload will be posted on the website along with the authors' responses. I agree for my report to be made available under an Open Access Creative Commons CC-BY license (http://creativecommons.org/licenses/by/4.0/). I understand that any comments which I do not wish to be included in my named report can be included as confidential comments to the editors, which will not be published.

Choose an item.

To further support our reviewers, we have joined with Publons, where you can gain additional credit to further highlight your hard work (see: https://publons.com/journal/530/gigascience). On publication of this paper, your review will be automatically added to Publons, you can then choose whether or not to claim your Publons credit. I understand this statement.

Yes Choose an item.
