# Supplementary material for: vEMstitch: an algorithm for fully automatic image stitching of volume electron microscopy
Source: Gigascience. 2024 Oct 26;13:giae076. doi: 10.1093/gigascience/giae076 (PMC11512480; doi:10.1093/gigascience/giae076)
Supplement: giae076_Reviewer_1_Report_Revision_1 — Yuelong Wu -- 7/8/2024 Reviewed [file giae076_reviewer_1_report_revision_1.pdf]

Reviewer Report

Title: vEMstitch: an algorithm for fully automatic image stitching of volume electron microscopy

Version: Revision 1 **Date:** 7/8/2024

Reviewer name: Yuelong Wu

Reviewer Comments to Author:

I am pleased to see that the authors have addressed one of my major concerns regarding the scalability of their tool. I appreciate their effort to re-implement their entire software in C++, and I thank them for specifying the focus of their work, steering away from applications like neural circuit reconstruction. Regarding scalability, could the authors also provide the runtime of the Python implantation of their tool alongside the C++ numbers? That information may help the potential adopters of this tool to make an informed decision between speed and convenience.I'm also glad to see the improved clarity of the manuscript. Following up on some of my other points, mostly just for clarifications:(2.1) Based on the authors' response, to clarify, the artifacts that rendered "accurate image stitching is almost impossible" were referring to the microscope issues, rather than those caused "during sample preparation" as stated in the manuscript text, correct? I also suggest the author to restrict the word "impossible" with something like "use only linear transformation model", given that a major point of this work is to use local elastic deformation to compensate for the imaging distortions. Could the author also elaborate the meaning of "radial damage" in their response? I couldn't find that term in the three references provided.(2.2) Thanks for the adding the empirical values for the coefficients, and it is reassuring to know a single set of settings work for most of the examples. Could the author response to my question regarding the selection of the window size? Does it enjoy similar robustness compared to the threshold coefficients.(2.3) Thanks for including the results that incorporated the prior knowledge of the overlapping ratio. I suggest adding markers to the curves in the new Fig 12 (a), as the orange curves corresponding to "filter isolated points" are hard to see in the precision panels for Experiment I & II. I'd also like to express my sympathy to the authors that they had to deal with unordered data.(2.6) Thanks for the clarification. So if I understand it correctly, the rigid parameters are estimated in a pairwise manner. Could the author provide information on how the local pairwise relationships are propagated to a global optimal solution? Would the authors expect error accumulation in such propagation process for larger sections?(2.7) Could the author elaborate why the granular appearance of the LoG filtered image poses challenges for later feature extractions?(2.8) Thanks for the additional experiment. So to clarify, in both ORB and SIFT experiments, FAST was used as the detector, then BRIEF and SIFT descriptors were used respectively, is that correct? Also could the author comment on why the direct SIFT method failed so catastrophically, while in the ablation test (suggested by Reviewer 2), results without local enhancement strategy turns out to be fine except for some local mismatches? Based on the structure of the manuscript, local enhancement happens during local deformation correction, why it seems to affect the rigid part of the transformation here? It seems in the direct SIFT experiment, the software did find ~7 correct matches, but it was some outliers that deviated the transformation. Did the authors control the filtering part and applied the same outlier strategy in these

experiments?Also, I'd like to follow up on the ablation test suggested by Reviewer 2. I fail to understand the authors' argument that ablation studies "have relatively weak explanatory ability" because the rigid part and elastic part are decoupled from each other, could the authors elaborate? I'd like to gently encourage the authors to conduct more thorough ablation experiments to investigate the effects of different components of the pipeline, such as outlier filtering, local enhancement, feature re-extraction and so on. This would provide the readers with a clearer picture of which treatments in the authors' method differentiate it from other established open-source tools.Finally, I'd like to point out that phase correlation based stitching tool (in the form of optical flow) that can handles elastic deformation also exists (e.g. https://github.com/google-research/sofima). It would be great if the authors could share their opinions on these tools compared to theirs.

Level of Interest

Please indicate how interesting you found the manuscript: Choose an item.

Quality of Written English

Please indicate the quality of language in the manuscript: Choose an item.

Declaration of Competing Interests

Please complete a declaration of competing interests, considering the following questions:

- Have you in the past five years received reimbursements, fees, funding, or salary from an organisation that may in any way gain or lose financially from the publication of this manuscript, either now or in the future?
- Do you hold any stocks or shares in an organisation that may in any way gain or lose financially from the publication of this manuscript, either now or in the future?
- Do you hold or are you currently applying for any patents relating to the content of the manuscript?
- Have you received reimbursements, fees, funding, or salary from an organization that holds or has applied for patents relating to the content of the manuscript?
- Do you have any other financial competing interests?
- Do you have any non-financial competing interests in relation to this paper?

If you can answer no to all of the above, write 'I declare that I have no competing interests' below. If your reply is yes to any, please give details below.

I declare that I have no competing interests

I agree to the open peer review policy of the journal. I understand that my name will be included on my report to the authors and, if the manuscript is accepted for publication, my named report including any

attachments I upload will be posted on the website along with the authors' responses. I agree for my report to be made available under an Open Access Creative Commons CC-BY license (http://creativecommons.org/licenses/by/4.0/). I understand that any comments which I do not wish to be included in my named report can be included as confidential comments to the editors, which will not be published.

Choose an item.

To further support our reviewers, we have joined with Publons, where you can gain additional credit to further highlight your hard work (see: https://publons.com/journal/530/gigascience). On publication of this paper, your review will be automatically added to Publons, you can then choose whether or not to claim your Publons credit. I understand this statement.

Yes Choose an item.
