# Supplementary material for: vEMstitch: an algorithm for fully automatic image stitching of volume electron microscopy
Source: Gigascience. 2024 Oct 26;13:giae076. doi: 10.1093/gigascience/giae076 (PMC11512480; doi:10.1093/gigascience/giae076)
Supplement: giae076_Reviewer_2_Report_Original_Submission — Erik C. Johnson, Ph.D -- 4/27/2024 Reviewed [file giae076_reviewer_2_report_original_submission.pdf]

Reviewer Report

Title: vEMstitch: an algorithm for fully automatic image stitching of volume electron microscopy

Version: Original Submission **Date:** 4/27/2024

Reviewer name: Erik C. Johnson, Ph.D.

Reviewer Comments to Author:

In the manuscript "vEMstitch: an algorithm for fully automatic image stitching of volume electron microscopy," the authors explore a new method for mixed global-local alignment and image stitching of volume electron microscopy images of biological tissue. This is an important and emerging area, with a new Volume EM community forming and large scale connectomics datasets being collected with electron microscopy techniques. Image alignment to create contiguous volumes is a necessary step before further annotation and analysis by machine or human analyst. There are several areas, including extended computational experiments, which could strengthen this manuscript for acceptance in GigaScience.Areas to address in a revised manuscript:* Introduction and background:1. In the introduction, it would be good to expand the literature and background survey on the emerging need for VolumeEM approaches. Of particular note is the emerging VolumeEM community, large EM connectomics projects and software infrastructure projects for data storage and analysis.2. It is also important to motivate the overall processing pipeline and problems. This includes sample preparation, imaging, pre-processing, segmentation/detection, manual analysis, etc. It is important to help the reader situate the image stitching problem within this bigger pipeline. Consider adding an introductory figure of a typical VolumeEM processing pipeline.3. As a related issue, it would be good to discuss the 3D alignment problem for VolumeEM, especially as it applies to techniques such as serial section EM. These 3D techniques work to merge image slices and create stacks in 3D, including global and local transformations. Some topics to consider in the context of this tool: A) Discuss isotropic (e.g. FIB SEM) vs anisotropic (e.g. serial section) imaging, and different needs for alignment and stitching to create contiguous volumes. B) Highlight differences and approaches to 2D stitching vs 3D alignment - what steps are required for each? What is similar between algorithms for these problems? C) Can vEMstitch be used in a 3D alignment pipeline or is it for 2D stitching problems only? D) Review and contrast against 3D alignment approaches to highlight advantages of vEMStitch, for example: Volume assembly process for the MICrONS dataset- Fig 2 of https://www.biorxiv.org/content/10.1101/2021.07.28.454025v3.full.pdf, Serial section EM pipelines https://www.frontiersin.org/articles/10.3389/fncir.2018.00094/full, https://github.com/AllenInstitute/em_coregistration*Results/Analysis/Computational Experiments:1. Table 1 should add significance testing relative to the new vEMstitch approach in addition to means/STDs, perhaps using a non-parametric test of difference in means.2. For approaches for stitching and alignment, it is critical to understand the impact of improved stitching or alignment on downstream processing and analysis. What is the impact of improved performance on the actual annotation/analysis questions that motivate VolumeEM? This is necessary to demonstrate some impact of improved stitching on downstream processing: A) Manuscript would be strengthened by an example of secondary

analysis using human or machine annotation on stitched images B)A useful example would be to apply segmentation (neuron segmentation) or object detection networks (synapse detection) to the CREMI data after stitching. Ground truth data is available for this - are detection/segmentation results improved with improved stitching? This will help demonstrate the impact of your new algorithm. This would need to be reported as a new table/figure. (Note: CREMI is a good choice for the controlled perturbation studies using subsamples as it is isotropic and contiguous, and ground truth annotations are available.)3. In order to better understand how the new pipeline works, additional ablation studies can be used to quantify performance (Note: Tables 3 and 4 were useful to gain insight into the parameters): For Table 1 - is it possible to have ablated versions of the pipeline (e.g. global only, different local elastic and blending settings) to understand the impact of each step on performance metrics- this would be key for motivating the multi-step algorithm. This could be included as a new table/figure.4. Computational complexity is a key issue when selecting algorithms for large volume EM, especially terabyte or petabyte image volumes: A) How does complexity compare between approaches? Is it possible to quantify with numerical experiments or computational complexity estimates? This could be included as a new table/figure. B) How does this scale with image tile size and/or overall volume size? Numerical examples would be best, but could also be discussed more generally.These additions would greatly strengthen the manuscript for a VolumeEM audience.

Level of Interest

Please indicate how interesting you found the manuscript: Choose an item.

Quality of Written English

Please indicate the quality of language in the manuscript: Choose an item.

Declaration of Competing Interests

Please complete a declaration of competing interests, considering the following questions:

- Have you in the past five years received reimbursements, fees, funding, or salary from an organisation that may in any way gain or lose financially from the publication of this manuscript, either now or in the future?
- Do you hold any stocks or shares in an organisation that may in any way gain or lose financially from the publication of this manuscript, either now or in the future?
- Do you hold or are you currently applying for any patents relating to the content of the manuscript?
- Have you received reimbursements, fees, funding, or salary from an organization that holds or has applied for patents relating to the content of the manuscript?
- Do you have any other financial competing interests?
- Do you have any non-financial competing interests in relation to this paper?

If you can answer no to all of the above, write 'I declare that I have no competing interests' below. If your reply is yes to any, please give details below.

I have no competing interests.

I agree to the open peer review policy of the journal. I understand that my name will be included on my report to the authors and, if the manuscript is accepted for publication, my named report including any attachments I upload will be posted on the website along with the authors' responses. I agree for my report to be made available under an Open Access Creative Commons CC-BY license (http://creativecommons.org/licenses/by/4.0/). I understand that any comments which I do not wish to be included in my named report can be included as confidential comments to the editors, which will not be published.

Choose an item.

To further support our reviewers, we have joined with Publons, where you can gain additional credit to further highlight your hard work (see: https://publons.com/journal/530/gigascience). On publication of this paper, your review will be automatically added to Publons, you can then choose whether or not to claim your Publons credit. I understand this statement.

Yes Choose an item.
