# Supplementary material for: vEMstitch: an algorithm for fully automatic image stitching of volume electron microscopy
Source: Gigascience. 2024 Oct 26;13:giae076. doi: 10.1093/gigascience/giae076 (PMC11512480; doi:10.1093/gigascience/giae076)
Supplement: giae076_Reviewer_2_Report_Revision_1 — Erik C. Johnson, Ph.D. -- 7/13/2024 Reviewed [file giae076_reviewer_2_report_revision_1.pdf]

Reviewer Report

Title: vEMstitch: an algorithm for fully automatic image stitching of volume electron microscopy

Version: Revision 1 **Date:** 7/13/2024

Reviewer name: Erik C. Johnson, Ph.D.

Reviewer Comments to Author:

Thank you for the detailed response and considerable improvement to the manuscript, including new subsections and figures.The manuscript makes an improved case for the efficacy of the vEMstitch tool and applicability to the volume EM community and their software workflows (including the impact on 3D reconstruction volumes).Some improvements could be made to the manuscript to address technical shortcomings and improve communication, however.1. The new experiments in section 3.2.2 are very useful. However, 1) be sure to introduce the Dice coefficient and Hausdorff distance and their interpretation. The experiments mention this is an average metric value- can standard deviations, and tests of population mean difference be added to Table 3? These details are critical.2. The key results are now scattered across several tables and figures, making it a challenge for the reader to find and assess the key claims. In my estimation, Table 1 and Table 2 along with Figure 8 and 10 make the key claimsa. vEMStitch outperforms phase-based alignment and critically TrackEM2 in your 3 experiments, b. computational complexity of vEMstitch is more comparable to Fiji and Mist, and c. You still see scaling with tile size and number of matching FPs comparable to TrakEM2Consider adding a first figure to the results section, to complement Tables 1 and 2, with a visual representation of the 3 experiments, visual comparison of the methods, and scatter plots of the runtime. This could consolidate images and data from the various tables and figures into one key figure to begin the results section. If redundant, later figures could be reduced or moved to the supplementary materials/appendix.3. Please carefully review the new section for editing and word choice, some examples"The similar numerical results across three experiments well demonstrate" -> The similar numerical results across three experiments demonstrate"" address different stitching problems, which are perhaps met in practice." -> " address different stitching problems, which are perhaps encountered in practice.""The corrected deformation filed helps to produce a clear mosaic of overlapping regions.""Both 2D stitching and 3D alignment are fundamental to the vEM pipeline, and they both aim to identify reliable patterns of two images" -> Both 2D stitching and 3D alignment are fundamental to the vEM pipeline, and they both aim to identify reliable patterns within two images"Thank you.

Level of Interest

Please indicate how interesting you found the manuscript: Choose an item.

Quality of Written English

Please indicate the quality of language in the manuscript: Choose an item.

Declaration of Competing Interests

Please complete a declaration of competing interests, considering the following questions:

- Have you in the past five years received reimbursements, fees, funding, or salary from an organisation that may in any way gain or lose financially from the publication of this manuscript, either now or in the future?
- Do you hold any stocks or shares in an organisation that may in any way gain or lose financially from the publication of this manuscript, either now or in the future?
- Do you hold or are you currently applying for any patents relating to the content of the manuscript?
- Have you received reimbursements, fees, funding, or salary from an organization that holds or has applied for patents relating to the content of the manuscript?
- Do you have any other financial competing interests?
- Do you have any non-financial competing interests in relation to this paper?

If you can answer no to all of the above, write 'I declare that I have no competing interests' below. If your reply is yes to any, please give details below.

I declare that I have no competing interests

I agree to the open peer review policy of the journal. I understand that my name will be included on my report to the authors and, if the manuscript is accepted for publication, my named report including any attachments I upload will be posted on the website along with the authors' responses. I agree for my report to be made available under an Open Access Creative Commons CC-BY license (http://creativecommons.org/licenses/by/4.0/). I understand that any comments which I do not wish to be included in my named report can be included as confidential comments to the editors, which will not be published.

Choose an item.

To further support our reviewers, we have joined with Publons, where you can gain additional credit to further highlight your hard work (see: https://publons.com/journal/530/gigascience). On publication of this paper, your review will be automatically added to Publons, you can then choose whether or not to claim your Publons credit. I understand this statement.

Yes Choose an item.
